# A dataset of annotated ground-based images for the development of contrail detection algorithms

**DOI:** 10.1016/j.dib.2025.111364

**Published:** 2025-02-04

**Authors:** Nicolas Gourgue, Olivier Boucher, Laurent Barthès

**Affiliations:** a Institut Pierre-Simon Laplace (IPSL), Sorbonne Université / CNRS, 4 place jussieu, Paris, France; b Laboratoire Atmosphères, Observations Spatiales (LATMOS), Université de Versailles-Saint-Quentin-en-Yvelines / Sorbonne Université / CNRS, 11 boulevard d'Alembert, Guyancourt, France

**Keywords:** Contrail, Ground-based camera, Hemispheric sky image, Polygon annotation, Aviation

## Abstract

All economic sectors must understand, measure and mitigate their contributions to climate change. The aviation sector is no exception and has to reduce its CO_2_ emissions while also addressing its non-CO_2_ effects which are responsible for a significant radiative impact on climate. The most important of these effects is due to the formation of contrails and their transformation into induced cirrus. Many studies have focused on detecting contrails onto satellite images because, taken together, meteorological geostationary and sun-synchronous satellites provide a good monitoring of the Earth's atmosphere, but unfortunately the spatial resolution and temporal sampling of such satellite images are often insufficient to detect contrails right after their formation and attribute a particular contrail to a given flight. The use of ground-based cameras, especially as part of a network, is therefore complementary to satellite imagery and currently represents an important avenue of research for contrail monitoring. In this article we describe a dataset of annotated ground-based hemispheric sky images that can serve as a basis for the training and validation of contrail detection algorithms, in particular those aiming at segmenting contrails using machine learning methods.

Specifications Table

**Table:** 

Subject	Earth and Planetary Sciences
Specific subject area	Atmospheric Sciences
Type of data	Annotated daytime RGB hemispheric sky images acquired by a ground-based camera. The images are pre-processed and annotated with labels and polygons.
Data collection	The images were acquired by a ground-based camera at the SIRTA [1] supersite in Palaiseau, France. The original RGB hemispheric images have a resolution of 1024×768 pixels but were reprojected onto a plane and truncated at 60° zenith angle. The images were then annotated and segmented manually using the Roboflow platform.
Data source location	The data were collected at the SIRTA observatory in Palaiseau (France), longitude 2.208°E, latitude 48.173°N, and are sampled throughout the year 2019.
Data accessibility	Data identification number: https://doi.org/10.57932/62c31b5f-ac28-4288-bd69-1cf8f07649b0 Direct URL to data: https://www.easydata.earth/#/public/metadata/62c31b5f-ac28-4288-bd69-1cf8f07649b0
Related research article	None

## 1 Value of the Data

• This data can be used to train and/or to validate contrail detection algorithms, in particular but not exclusively those using neural network architectures.
• This dataset enables to study the formation of non-persistent and persistent contrails up to a few minutes. These time sequences can be used to develop and validate neural networks to segment contrails throughout their lifetime in the field of view of the camera.
• Polygon annotation allows for a precise segmentation of contrails.
• The dataset spans a wide range of illumination and meteorological conditions, especially in terms of cloud cover.
• Data produced by ground-based cameras complement the satellite approach to improve attribution of a contrail to a particular aircraft and determine with high frequency when and where contrails are produced.

## 2 Background

Contrails are white streaks that form behind aircraft at cruising altitudes when the atmosphere is cold and wet enough. They are mainly composed of ice crystals and can evolve towards cirrus clouds when the atmosphere is supersaturated with respect to ice. Contrails and induced cirrus interact with solar and terrestrial radiation and are responsible for a positive radiative forcing, thus contributing to warm the climate system. It is therefore important to better understand their formation and their evolution in order to propose mitigation measures to the aviation sector [2]. Sky images, whether taken from below (i.e., from ground-based cameras) or from the top (i.e., from satellites), represent an invaluable resource to observe contrails. However, large amount of data are needed to sample the large variety in atmospheric conditions. Artificial Intelligence (AI) methods make it possible to process such large quantities of data in order to generalize the results. However supervised machine learning (ML) methods require high-quality, annotated data upon which they can be trained and validated. While there are several studies segmenting contrails in satellite images [3], [4], there are few open datasets of images with contrails taken from the ground. The OpenContrails dataset [5] is a reference in the field, but it only contains satellite images, as is the Landsat-8 contrails [6] dataset. We feel it is essential to complement this dataset with images from ground-based cameras because meteorological satellites have limitations in terms of spatial resolution and/or temporal sampling. Indeed their resolution is generally too low to observe the formation phase of contrails which can only be detected after a few tens of minutes [3]. Ground-based cameras, in contrast, allow the detection of contrails almost instantaneously after their formation, thanks to their contrast and resolution, making it possible to attribute a contrail to a particular flight and analyze formation conditions more precisely [7], [8], [9]. Pertino et al. [10] have used RGB and infrared (IR) images from ground-based cameras to complement the satellite approach. They showed the feasibility of using computer vision models to detect contrails but their database is relatively small and their annotated dataset is not open source. In this context we present here an annotated open-source dataset of ground-based sky images from a hemispheric camera.

## 3 Data Description

### 3.1 Image types

**Fig. 1:**
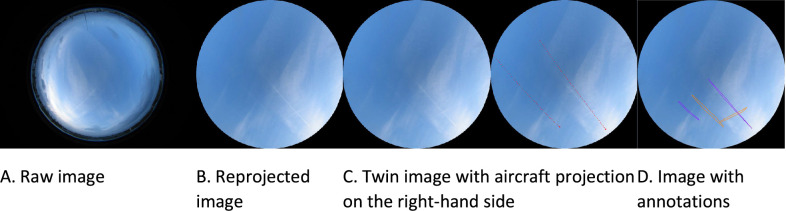
The four different types of images provided in the dataset.

The dataset provides four different types of images for 1600 scenes. Fig. 1 shows an example of the four different image types. The raw image (Fig. 1A) corresponds to the default image provided by the hemispheric camera of the SIRTA site. It has 1024 by 768 pixels with RGB channels and provides a hemispherical view of the sky, thanks to a fish-eye lens, surrounded by black areas. We carried out four pre-processing steps to generate the second type of images (Fig. 1B): removal of the black strips on both sides of the image disc, removal of the pixels located beyond a zenith angle of 60°, reprojection of the image using an arctan function to restore the straight lines, and flip of the image along the vertical axis so that when viewed with imshow function in python, the top of the image corresponds to the north and right side to the east. In the third image type (Fig. 1C), we duplicate the image and produce twin images so that the left image can be used for the annotation with the help of the right image that also shows the projected aircraft positions (red squares) and their trajectories (dotted red segments). We use coincident ADSB data acquired at the SIRTA site to determine the real-world aircraft positions. ECMWF Reanalysis v5 (known as ERA5) data are used to compute the vertical temperature profile and convert the barometric altitude provided by the ADSB signal into a geometric altitude. The longitude, latitude and geometric altitude can then be projected onto the image (see Methods in Section 4). The aircraft trajectories correspond to the aircraft positions during the three minutes preceding the acquisition time of the image. The fourth type of images (Fig. 1D) is for the annotated images with seven categories of annotations: four contrail classes and three ancillary classes, as described in the next subsection. We supply the images in .jpg format and a .json file containing the annotations.

### 3.2 Annotation of classes of objects

**Fig. 2:**
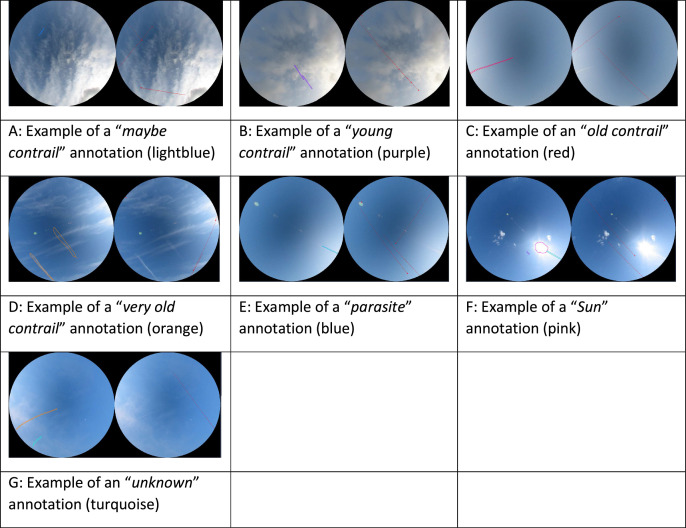
Examples of annotations for the 7 classes of objects.

We now describe the annotated objects (refer to Fig. 2 for examples). The first class of contrails (Fig. 2A) is labelled as “*maybe contrail*” corresponding to short trails with very little contrast that may or may not be a contrail. These objects are usually very small and are only annotated because they are close to the aircraft trajectory and could therefore correspond to very-short lived contrails. The second class (Fig 2B), labelled as “*young contrail*”, correspond to unambiguous condensation trails that appear in the image next to an aircraft trajectory and within a three-minute period after the aircraft has passed in the camera's field of view. Note that three minutes also correspond to the typical time it takes an aircraft to cross the image. It should be noted that some aircraft do not emit an ADS-B signal so it is not unusual to see a very young contrail without an ADS-B track nearby. We do annotate such contrails if the image sequence confirms their sudden appearance in one image and their advection or evaporation in the following images. The third class (Fig. 2C), called “*old contrail*”, identify contrails for which the aircraft has been out of the image field for more than three minutes, but which are still rectilinear. The fourth class of contrail is labelled as “*very old contrail*” (Fig 2D). These contrails are sufficiently old for the wind to have deformed them, and generally have heterogeneous opacity.

The other three classes of objects are labelled “*parasite”* (Fig. 2E), *“Sun”* (Fig. 2F), and “*unknown”* (Fig. 2G). Parasites are usually reflections of the Sun that are straight and white and could be mistaken for contrails. A simple way to tell the difference between the two is to compare the image being annotated with the previous or next one in time. While actual contrails move with the wind, reflections due to the Sun are stationary. For the “*Sun”* class, we annotate the bright, saturated area around the Sun but not the rays that may expand radially from the Sun disc. The *“unknown”* class correspond to cloud streaks that have generally formed outside the camera's field of view and are thus not easily attributable to a contrail even when rewinding in time the original, non-truncated images. Finally, to facilitate the segmentation process in ML methods, we added two classes, namely the “*Sky*” and “*Background*” classes. The sky class corresponds to the background sky of the image, whether blue sky or cloudy while the background class corresponds to the black background of the image in the four corners of the square. All the classes are summarized in Table 1.

**Table 1:** List of classes used in this study and their characteristics.

Class of object	Description
"*Maybe contrail*"	An aircraft trajectory is present. The object looks like a *young contrail* but has very low contrast.
“*Young contrail*”	An aircraft trajectory is present. A contrail object can be unambiguously matched to the trajectory. The contrail is less than 3 minutes old.
“*Old contrail*”	The aircraft trajectory has left the image. The contrail object is still linear but is more than 3 minutes old.
“*Very old contrail*”	The aircraft is well outside the image. The contrail object is no longer linear and may be heterogeneous. The contrail is likely to be more than 10 to 15 minutes old.
“*Parasite*”	The object is a feature that may look like an *old contrail* but that does not move with the wind when looking at successive images.
“*Sun*”	The object corresponds to the pixels saturated by the Sun but is limited to a disc and does not include sunrays. A Sun object hidden by a cloud is not annotated.
“*Unknown*”	The feature is a cloud streak that may look like an *old contrail* or a *very old contrail* but we cannot determine whether it is a contrail or a natural cirrus.
“*Background*”	The black pixels in the four corners of the image.
“*Sky*”	All other pixels in the image.

### 3.3 Statistics

**Fig. 3:**
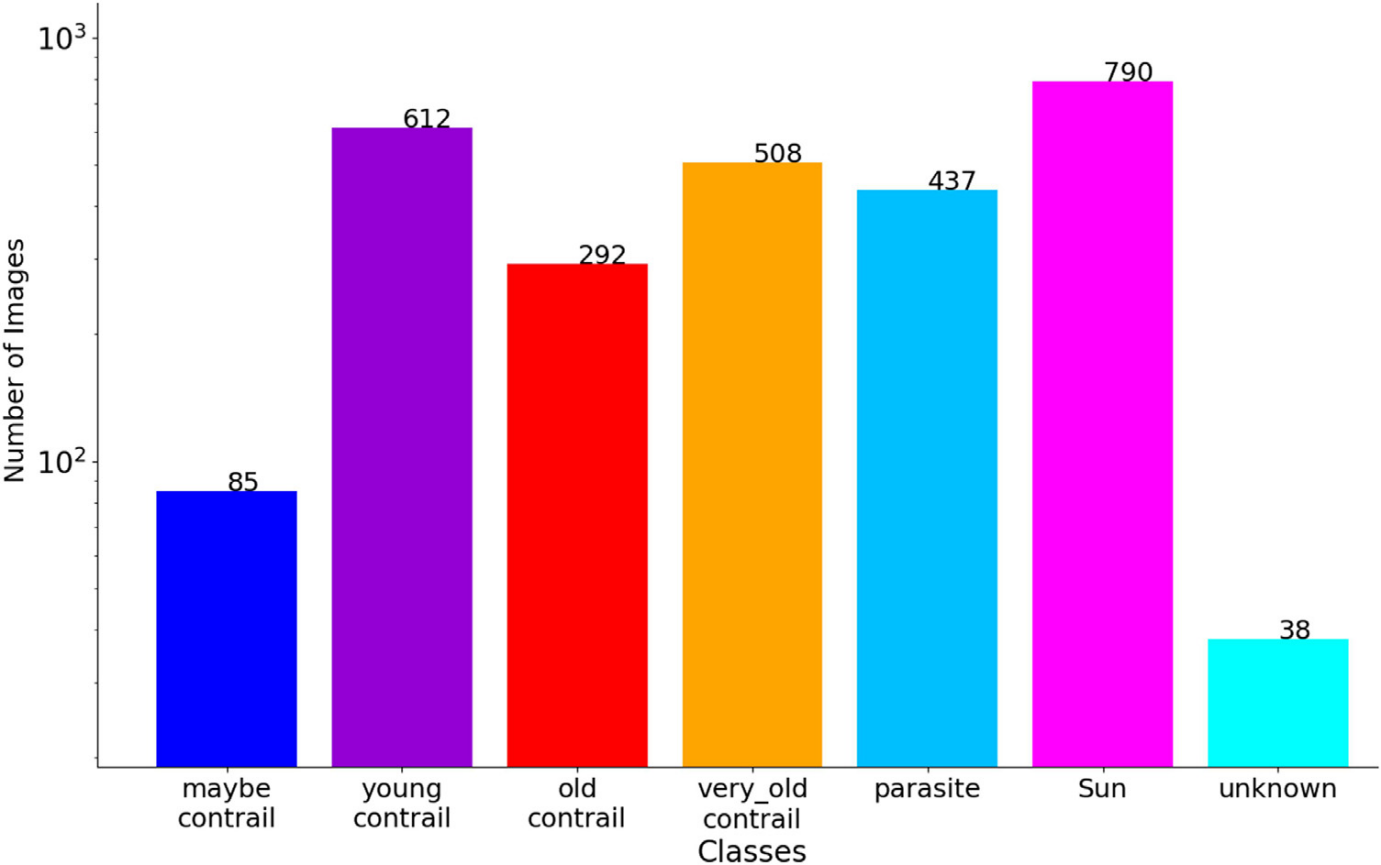
Histogram of the number of images with at least one annotation from a given class. The total is larger than the number of annotated images as one image can have annotations from several classes. Note the logarithmic scale on the y-axis.

Fig. 3 shows the number of images by class for the whole dataset. Images with the “*maybe contrail*” class are relatively few in number, as they are not visible on successive frames (otherwise they would be classified differently). Older wisps are not very present in the images, as they are only present in one or two images maximum per aircraft trajectory. In the case of persistent contrails, an image with a “*young contrail*” is associated with 3 or 4 images with an “*old contrail*”, and 5 to 8 images with a “*very old contrail*”, which is due to the natural evolution of the contrail. To counterbalance this, we have selected a larger number of images with non-persistent contrails, as this allows us to have *young contrail*s with no older contrails. *Parasite*s only occur on certain sunny days, but tend to persist as long as the Sun is present in the image. It is therefore normal from them to come in smaller number than the Sun. These two classes are also very present, as the days of February 6 and 9 were annotated completely, so there is a number of images that had no streaks but *parasite*s and the Sun. The unknown category can be considered as the scrap class. It groups together objects that resemble either an old or a very old contrail. When annotating, we proceeded in two stages. First, we annotate all the objects corresponding to the different classes. But we classify as indeterminate all objects that have formed outside the camera's field of view and do not behave like a classic “*old*” or “*very old contrail*”. However, in a second step, we reconsidered the annotation by examining the temporal dynamics based on the previous and next images to see the evolution of the object, looking at both the raw and preprocessed images.

**Fig. 4:**
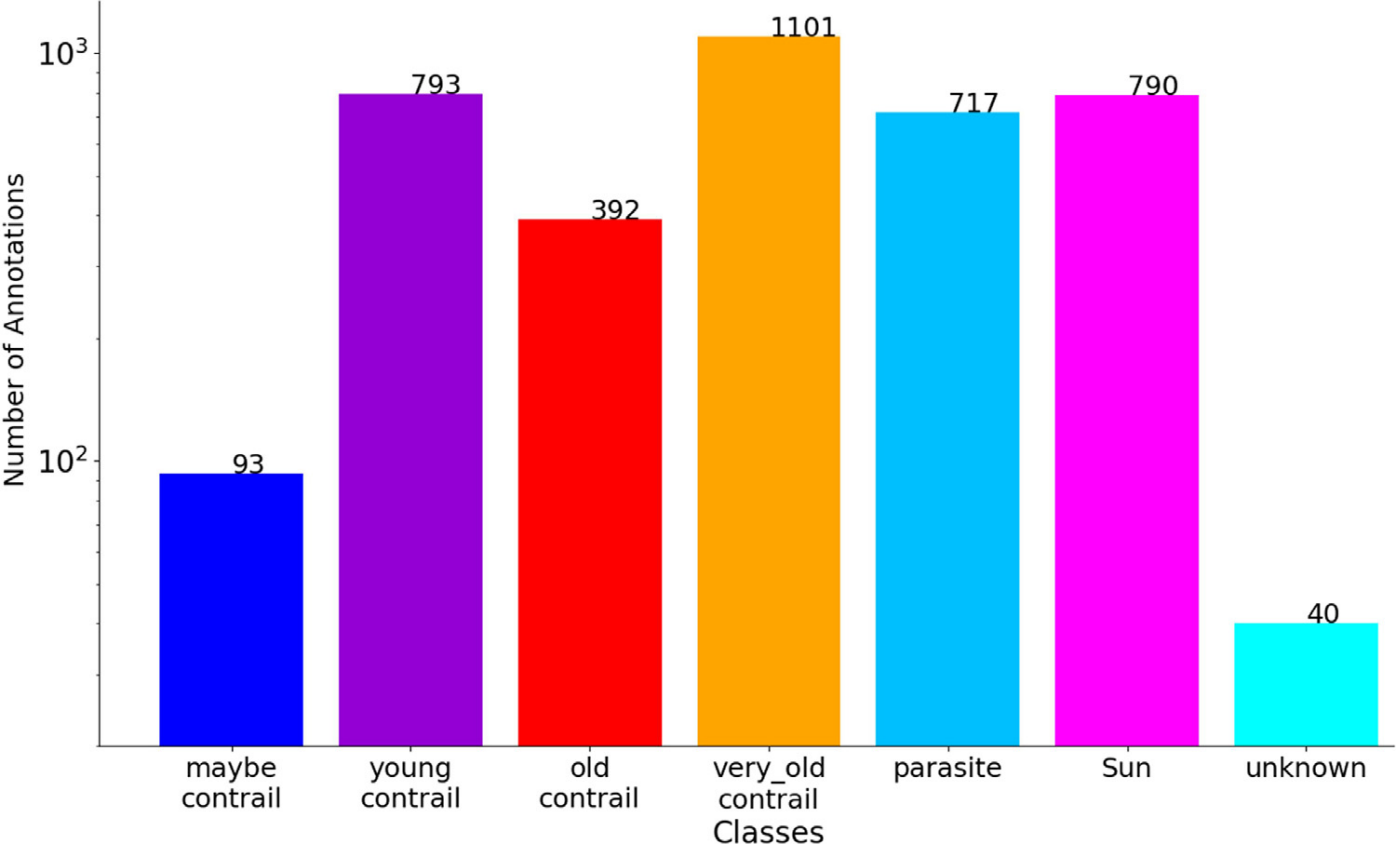
Histogram of the number of annotations from a given class. Note the logarithmic scale on the y-axis.

Fig. 4 is a variant of Fig. 3 where we show the number of objects in the classes rather than the number of images with the classes. Apart from the “*Sun”* class, which does not change because there is at most one Sun per image, the other classes show larger occurrences but maintain the general trend. There is therefore no significant disparity between the number of images showing the different classes and the number of objects in the classes.

**Fig. 5:**
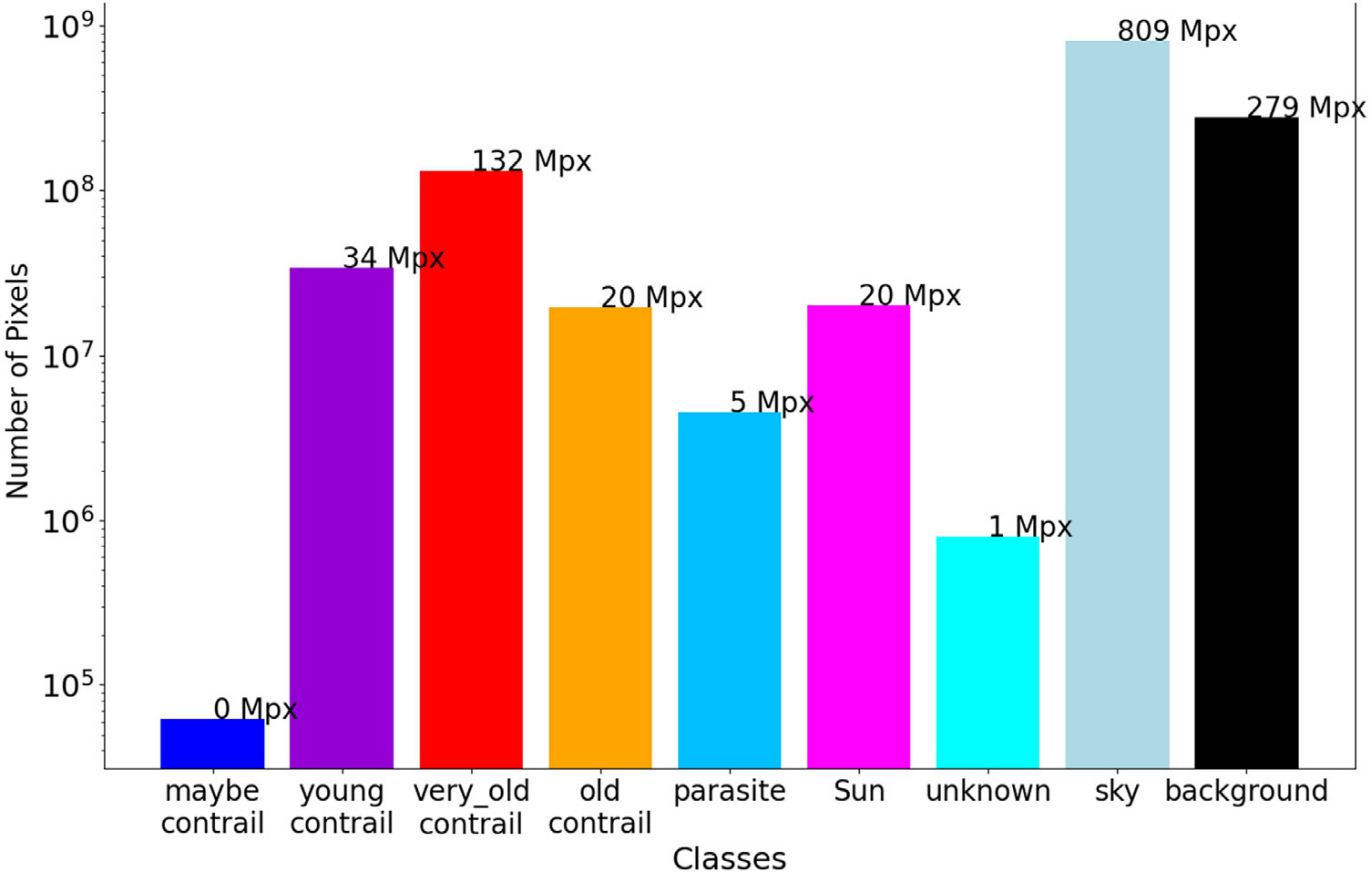
Histogram of the number of pixels for a given class. The Sky and Background pixels have been considered as well for comparison. Note the logarithmic scale on the y-axis.

When looking at the number of pixels per class (Fig. 5), the classes become more unbalanced. The “*sky*” and “background” classes largely dominate due to the small size of the contrails compared to that of the background. It is interesting to note that although the number of “*young contrail*” objects is of the same order of magnitude as that of “*old contrail*” and “*very old contrail*”, this is not the case in terms of number of pixels. Indeed, as time passes, contrails spread out leading to an increase in their surface area. Thus, the pixels flagged as “*very old contrail*” are almost seven times more numerous than those flagged as “*young contrail*”.

### 3.4 Annotation of tags

Our classes (Table 1) that characterize the images at the pixel level are supplemented by tags (Table 2). These tags can be used to classify images according to cloud cover, the presence of cirrus clouds, the presence of rain or snow, or the presence of circular reflections of the Sun on the image. A “complex” tag means that the image contains a large number of contrails of varying ages and that annotation of the image may not be reliable. The full list of tags is as follows:

**Table 2:** List of tags used in this study.

Tags	Description of tag
**Cloud Cover**	
Blue Sky	There is no visible cloud on the image.
Broken Cloud	Less than ∼50% of image is cloudy.
Mostly cloudy	More than ∼50% of image is cloudy.
Full overcast	The image is completely cloudy. This also includes images with semi-transparent cirrus.
**Other Tags**	
Artefact	The lens reflection produces circle with green or red flashes.
Cirrus	A cirrus cloud (with some degree of transparency) is present on the image.
No contrail	The image is without contrail.
Day complete	The image belongs to a day for which all images were annotated (i.e., 06/02 and 09/02/2019).
Complex	The image has many objects so it was difficult to annotate correctly and some objects may be missing.

**Fig. 6:**
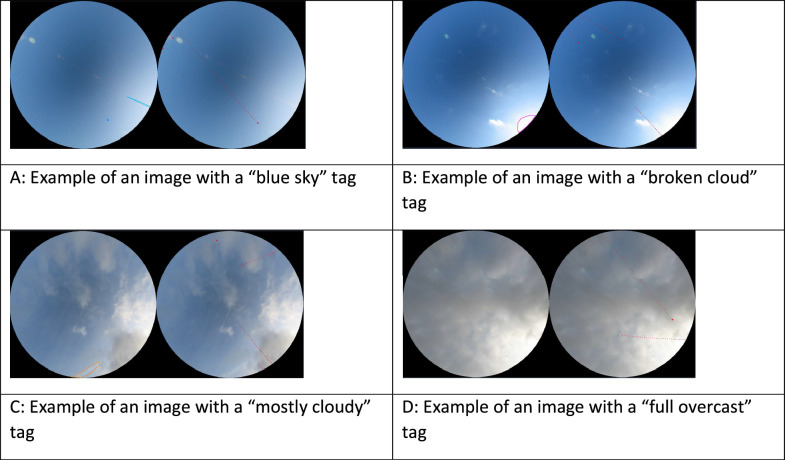
Examples of images with the different cloud cover tags.

**Fig. 7:**
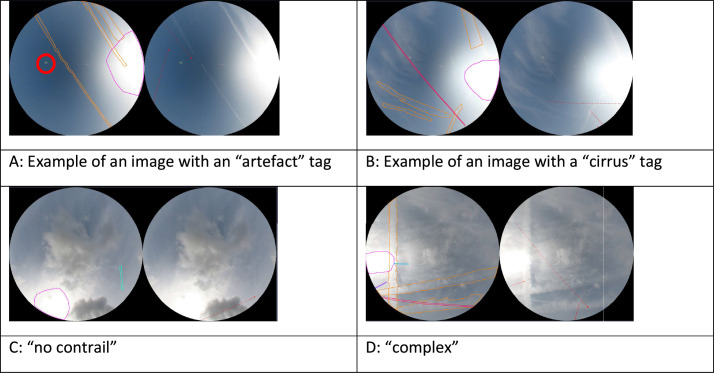
Examples of images with ”artefact”, “cirrus”, “no contrail” and “complex” tags. Note that on example A, the artefact is circled with a thick line but this does not correspond to an annotated object.

Fig. 6 illustrates the four tags that relate to cloud cover, namely “blue sky”, “broken cloud”, “mostly cloudy” and “full overcast”. We complement this information with a “cirrus” tag that indicates the presence of semi-transparent clouds as shown in Fig. 7B.

The “artefact” tag (Fig. 7A) is used to indicate that a large area in the axis of the Sun is very bright or saturated. The “no contrail” tag (Fig. 7C) is added to easily reject images not containing any contrail, irrespectively of the cloud cover tag. The “no contrail” tag does include “maybe contrail”. An image with only “maybe contrail” is not considered to have contrails and is therefore tagged no-contrail. Finally the “complex” tag (Fig. 7D) corresponds to images that are very busy and therefore difficult to annotate. The “cirrus” tag (Fig. 7B) refers to images where the cloud is partially transparent, thus contrails may be visible despite the cloud cover. The “day complete” tag concerns images where the day has been annotated in its entirety, regardless of the presence of contrails or not. This applies to February 6 and 9, 2019.

**Fig. 8:**
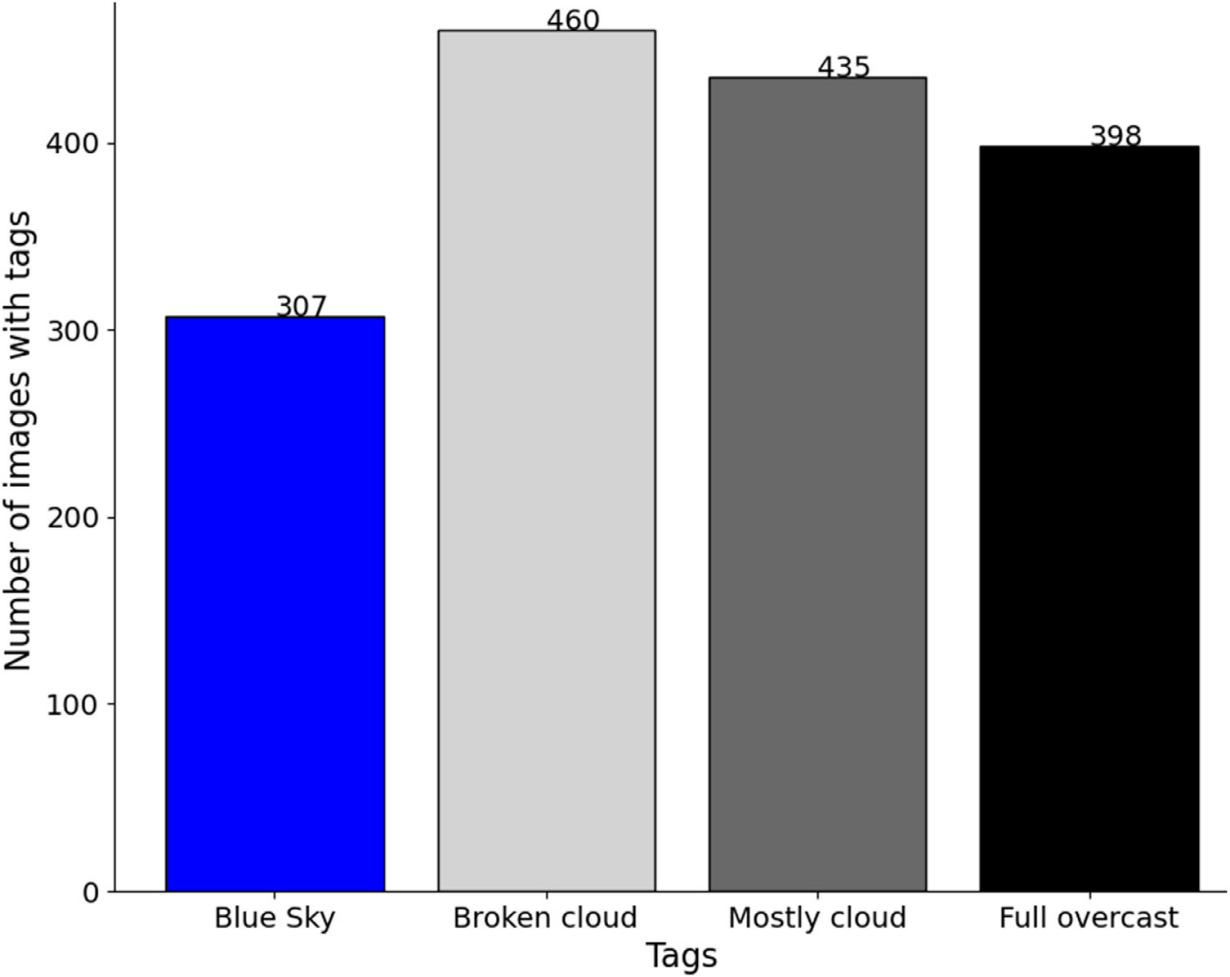
Histogram of the number of images as a function of the cloud cover tags.

The number of cloud cover tags (Fig. 8) is close to be balanced across the dataset because we purposely chose images with different cloud covers. “*Blue sky”* conditions are in general less favorable to persistent contrails. That is why the “*blue sky”* tag is less present. In each cloud cover condition, we tried to have a minimum number of images with and without semi-transparent cirrus clouds. Images with the “*full overcast*” tag are not necessarily without any class object as these images may show contrails on top of semi-transparent cirrus clouds. Only images with the “*full overcast*” label and without the “*cirrus”* label do not have any contrail or “*Sun*” objects in a systematic way.

**Fig. 9:**
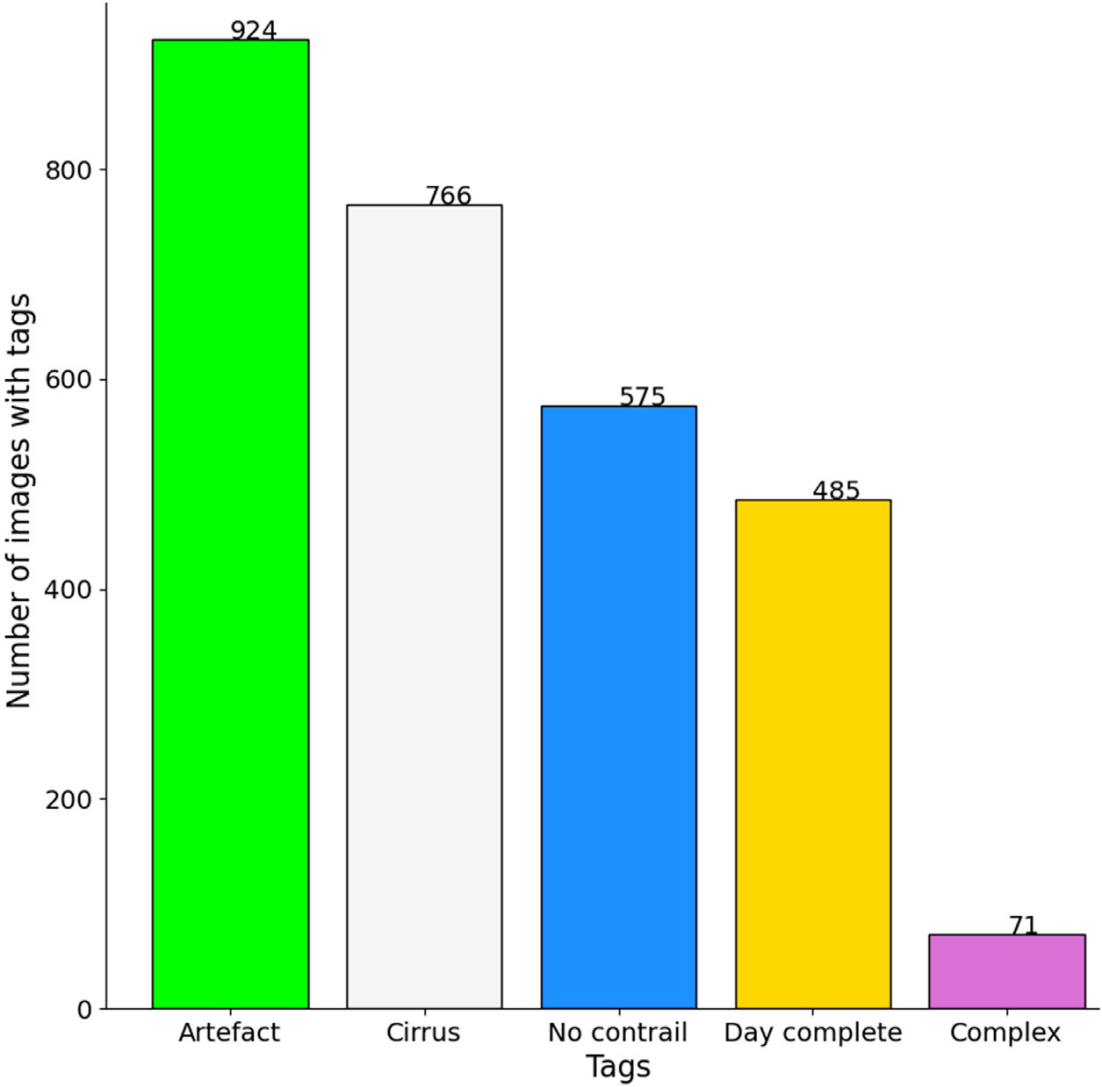
Histogram of the number of images with the other tags.

In addition, the dataset contains 924 images with an “artefact” tag in Fig. 9, representing 58% of the total number of images and 100% of the blue sky images when the Sun is visible. We also tried to have a fair distribution of “cirrus” tags, with 766 out of 1600 images. Images with the “no contrail” tag come mainly from the two fully annotated days and from a few cases with “*maybe contrails”*.

As mentioned above, the dataset consists of 1600 images taken in 2019. We sampled the images as follows. First we selected random dates in each month. After January we had enough cirrus-free full overcast sky images. Then we continued to draw random days per month until the end of July. As at the end of the year, we were missing certain weather condition combinations, such as artefact-free blue sky images (generally present at sunrise and sunset), we went through each day backward from the end of the year until we had enough images. The aim of this sampling was not meant to have a faithful distributions of images across the year, but rather a relatively balanced diversity of cases. This is justified by the fact that the main purpose of this database is to be used for automatic learning, which requires, as a general rule, a balanced image database so that the neural network can “see” the different cases. To this end, the sampling strategy we have chosen ensures that many weather situations are represented in the dataset along with different types of contrails.

### 3.5 Seasonal and daily cycles

**Fig. 10:**
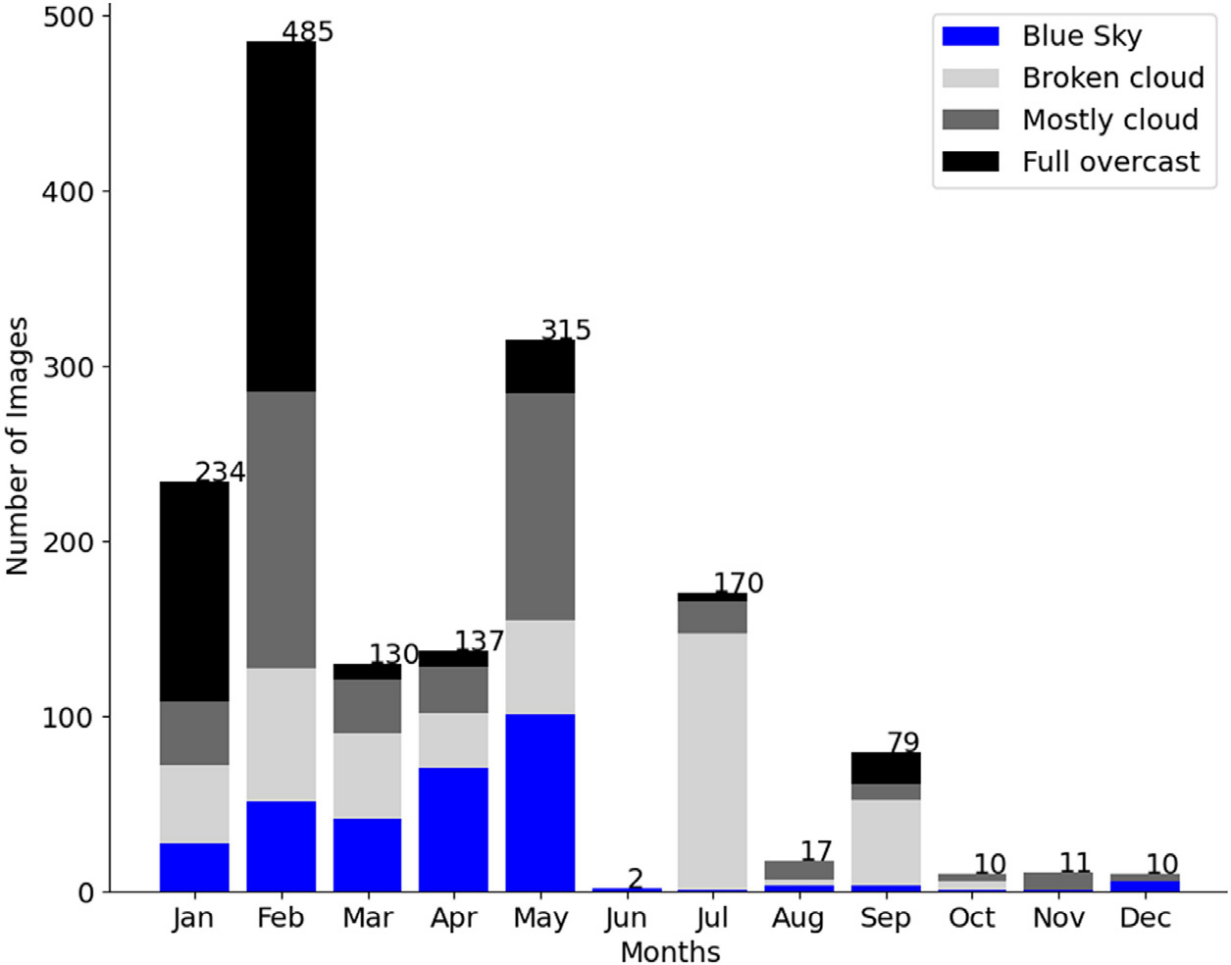
Histogram of the number of images (and its repartition between the four cloud cover tags) as a function of the month in the year.

The database has two time dimensions characterized by the day in the year (seasonal cycle) and the hour in the day (daily cycle). A distinction must be made between the daily sampling, which results from the presence or absence of contrails, and the seasonal sampling, where the days have been partially selected at the beginning of the year and as the year progresses in order to balance the cloud cover conditions (Fig. 8). We can also see in Fig. 10 that in the first five months the different proportions of cloud cover are of the same order of magnitude (except for “Full overcast”). As before, we compare the histograms for the number of images (Fig. 10) with those for the numbers of objects and pixels (Figs. S1 and S2). There is no significant difference between the three histograms as the proportions of the different objects are conserved.

**Fig. 11:**
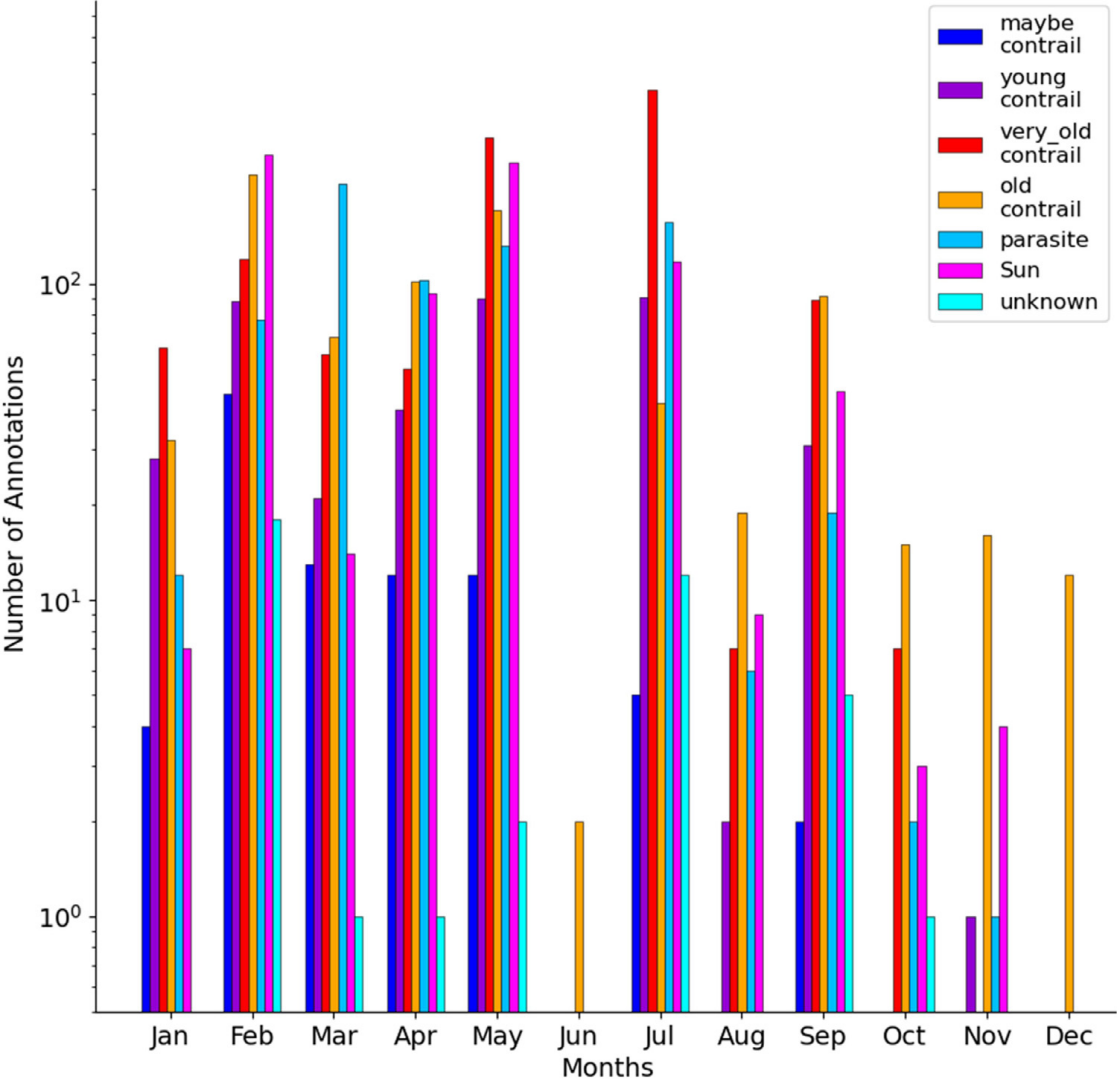
Histogram of the number of annotations by annotation class and as a function of the month.

In terms of annotation classes, we get a similar repartition of images across the months from January to July, with the exception of June (Fig. 11). As annotations are much lower at the end of the year, class ratios are no longer retained. The changes in representation are not explainable by the weather conditions but rather results from our sampling strategy. Please refer to Figs. S3 and S4 for the statistics on the number of objects and pixels.

**Fig. 12:**
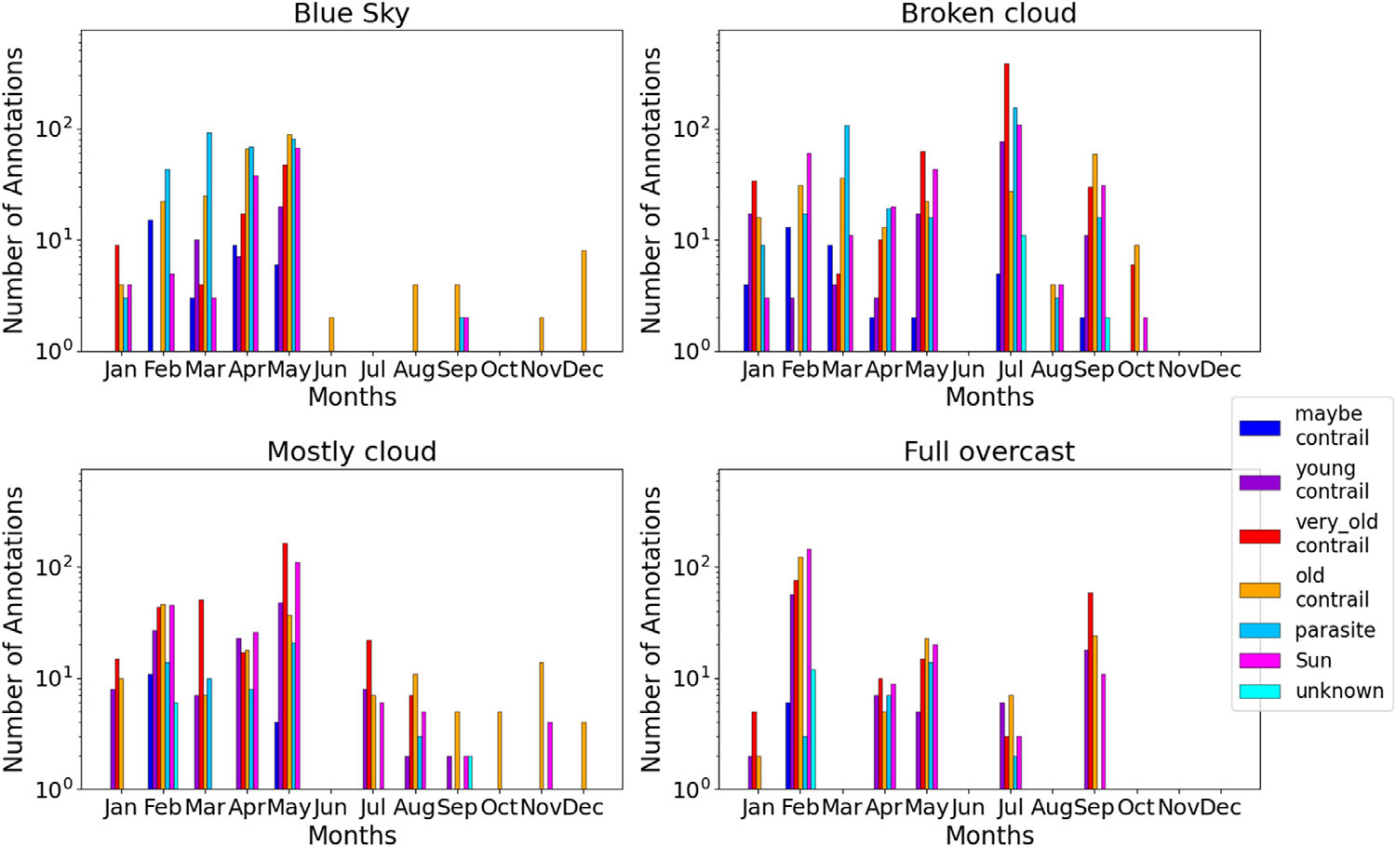
Repartition of annotation by class (color scale) and cloud cover (top left: “blue Sky”; top right: “broken cloud”; bottom left: “mostly cloudy”; bottom right: “full overcast”).

We show on Fig. 12 the repartition of the annotation classes for each cloud cover tag. The broken cloud cover appears to have the best annual balance. The full overcast class experiences all different contrail classes but only for some months. Fig S5, 56 seem to show the same characteristics for images and pixels.

**Fig. 13:**
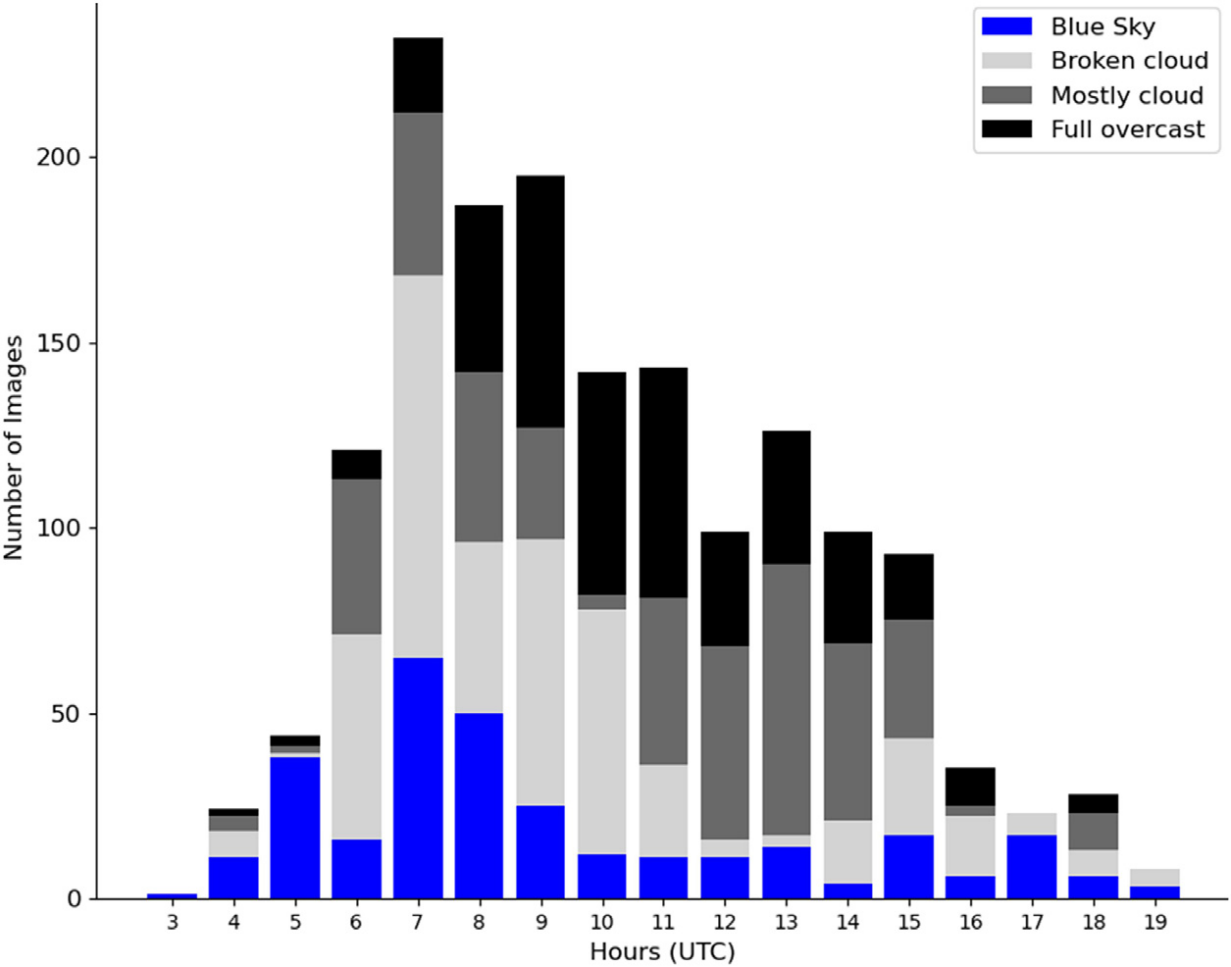
Histogram of the number of cloud cover tags as a function of the hour (UTC) in the day.

We are now interested in the analysis of the daily cycle. The under-representation of early and late hours (Fig. 13, S7, S8) is explained by the course of the Sun in the sky across the seasons as we only consider images from sunrise to sunset. The morning peak of contrails highlights the highest occurrence of contrails in the morning in accordance with the literature on the subject [7]. It is also explained by the need to balance the dataset across the different contrail tags.

**Fig. 14:**
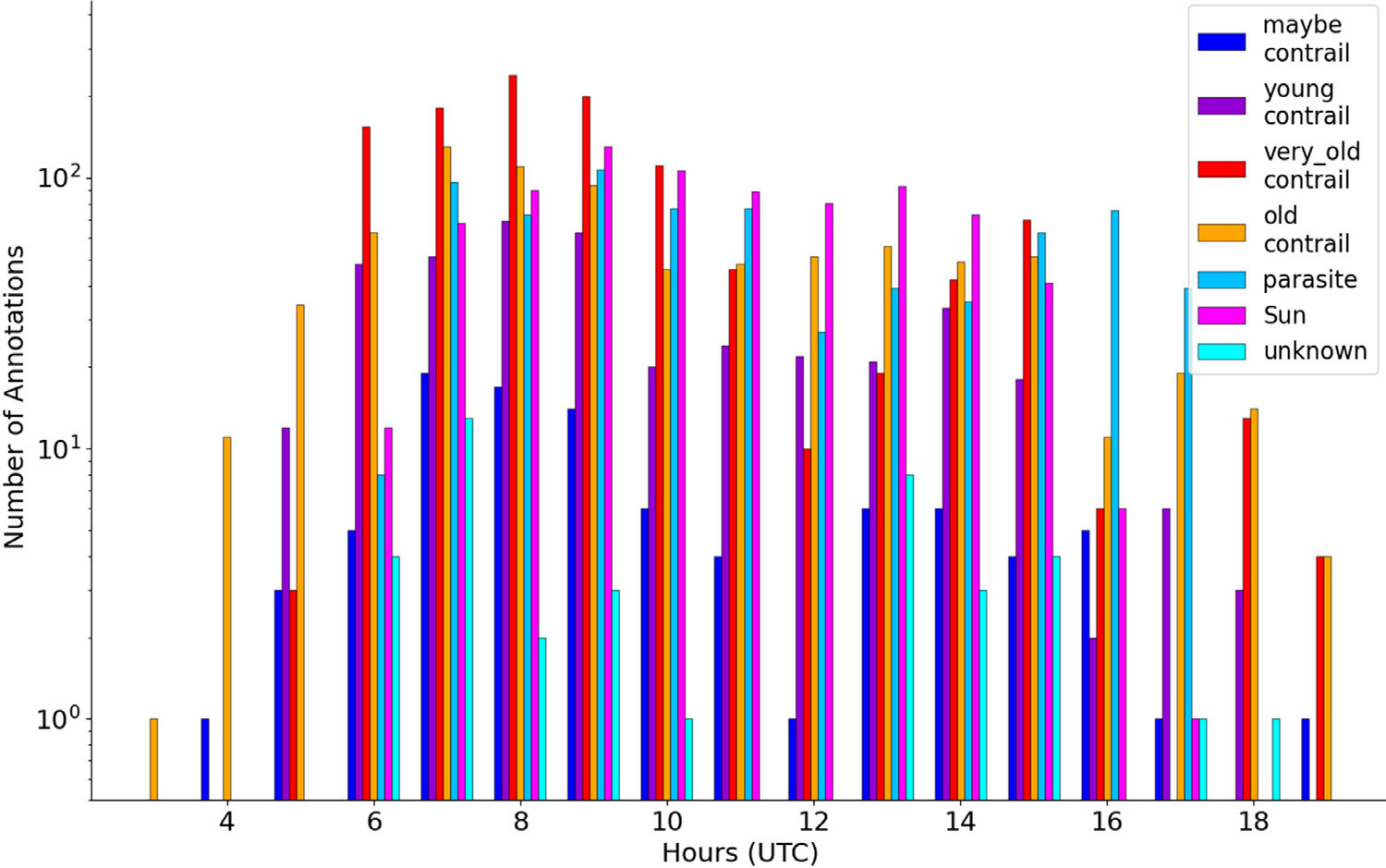
Histogram of the classes as a function of the hour in the day.

Fig. 14 shows the histogram of the classes of objects at different times of the day. The same morning peak as in Fig. 13 can be seen. *Parasite*s do not seem to have a preferred time of day to appear. Figures S9 and S10 show that there is no major discrepancy between the representations of objects, images and pixels for the time cycle per class.

**Fig. 15:**
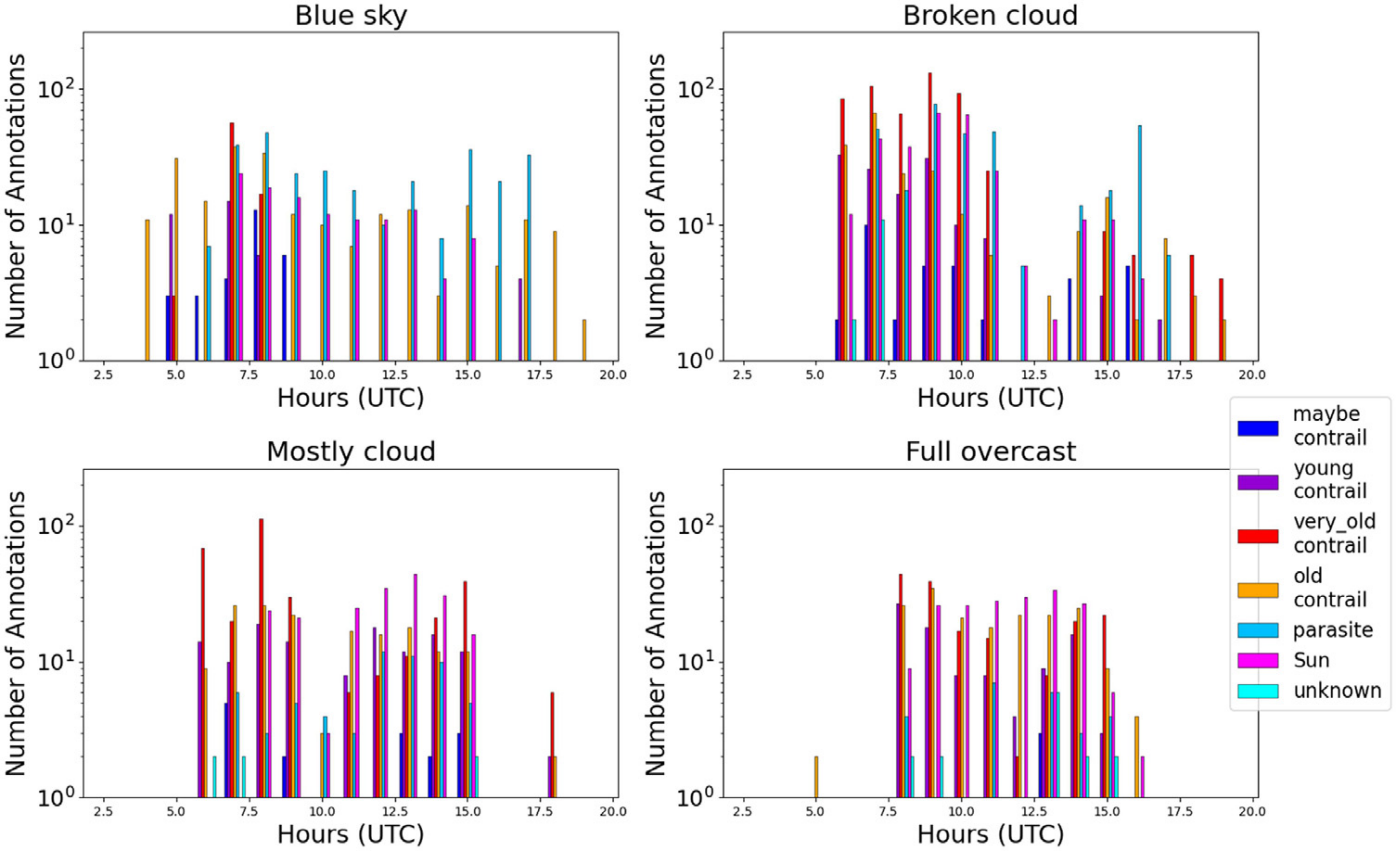
Repartition of annotations by class of objects and cloud cover (top left: “blue Sky”; top right: “broken cloud”; bottom left: “mostly cloudy”; bottom right: “full overcast”)

When looking at the histograms of annotations by cloud cover classes (Fig. 15, S11, S12), it can be observed that the Sun becomes more frequent with increasing cloud cover. The “*young contrail*s” class dominates in “blue sky” conditions, which can be explained by more favorable conditions for spotting small objects. In contrast the “very old contrails” class dominates in cloudy conditions as contrail persistence is also favorable to cirrus formation.

### 3.6 Pixel wise density of classes

**Fig. 16:**
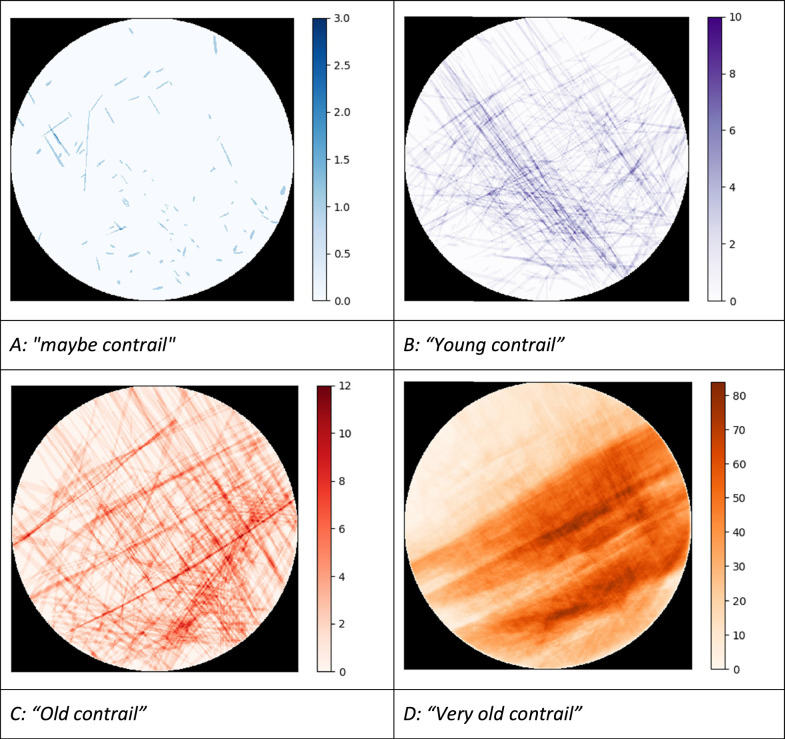

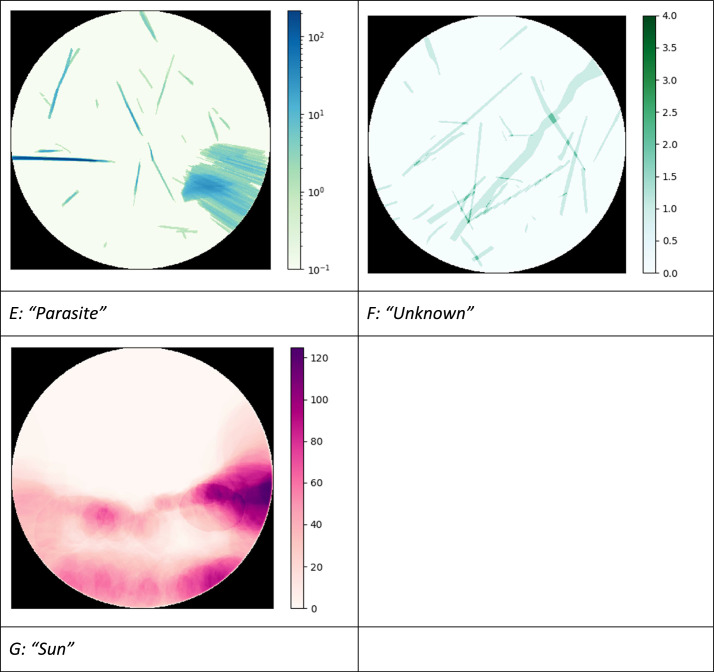
Composite figure showing the number of pixels annotated for each class across the dataset.

Fig. 16 shows for each pixel how many times it was labelled with the corresponding class across the entire dataset. The “*Sun”* composite image (Fig. 16G) confirms the East-West course of the Sun and the slightly imbalance in the selection of the months as well as morning conditions being more prone to clear-sky conditions and contrail detection. As for ``*maybe contrail*s'' (Fig. 16A), there does not seem to be any privileged areas. For *“young contrail*s” (Fig. 16B), the direction from top left to bottom right seems to be preferred. But the persistent streaks (Fig. 16C and D) are oriented more often from lower left to upper right. This implies that contrails that do not persist do not have the same orientation as those that do persist, which could be due to different aircraft routes occurring at different altitudes and the limited sampling of meteorological conditions. Fig. 16E, for parasites, is in log scale, as the values on the right-hand side of the image are very high. It appears that the camera lens has been stained over time, leaving a trace in this area, while the rest of the camera seems less prone to this. The lower-right area also appears to have reflections, but the fact that they change with the sunlight seems more indicative of a random soiling rather than a genuine staining. As far as “*unknowns”* are concerned, we have very few cases but they seem to favor the same direction as the old and very old contrails so we cannot rule out that they have a contrail origin.

## 4 Experimental Design, Materials and Methods

Images (Fig. 1A) are acquired using an Eko camera positioned at the SIRTA [1] observatory, in Palaiseau (France). This camera is equipped with a Cmos sensor with a resolution of 1024×768 pixels for a circular image of 678 pixels. The camera is equipped with a fish-eye lens, giving a hemispherical view of the sky. Images have been acquired every 2 minutes from sunset to sunrise. We concentrated the annotation on images sampled in the year 2019, during this year the camera was sealed onto a stable pillar.

These images are pre-processed in Python using the skimage library to remove the black strips and to re-project them onto a horizontal plane assuming the pixels are distributed linearly with the zenith angle outwards from the centre of the image. This results in a 901×901 image (Fig. 1B), so that to keep the same resolution at the center of the image as in the original image. To remove the various landscape artefacts, we cut the camera view at 60° zenith angle.

We could then perform an angular calibration of the images which associates a direction (or a point on a sphere with unit radius) in the real space to an image pixel and vice versa. For this we use the angular model of Jeanne et al. [11]. The free parameters of this model were calibrated in a previous work so that i) the centers of the Sun discs match the reprojected positions of the Sun on the images and ii) fresh contrails are aligned with their respective reprojected aircraft positions on the images.

For the annotation, we projected the position of the aircraft onto the image using an Automatic Dependent Surveillance-Broadcast receiver (ADS-B) signal and duplicated the image to obtain a version with aircraft trajectories and one without (Fig. 1C). The aircraft trajectories are projected for the 3 minutes preceding the image acquisition. The annotations are made on the image without the projected aircraft positions. The database includes the annotated part of these twin images (Fig. 1D).

The image files contain some metadata, such as the exact time the image was taken.

## Limitations

We identified a number of limitations for this dataset. The first limitation is the amount of images that only contain 1025 contrails. However, this limitation can be partially mitigated by data augmentation before or during the training phase of machine learning algorithms. The second limitation relates to the robustness of the annotation process. All the images were annotated by a single person, which means that quality control is not possible. However, each image has been examined several times and, in many cases, the previous and next images were also examined before settling on a class or a tag. Furthermore we requested some feedback from the members who went through the database and provided feedback that led to a number of corrections being made to the database. Finally, as the camera's field of view is quite small, contrails can only be tracked during about 10 minutes during windy periods but up to 30 minutes in less windy conditions.

It should be noted that there are successive images for some given dates. Hence, if this dataset is used to train and validate deep learning contrail detection algorithm, it will be important to define adequately the training, validation and test datasets so that they cover disjoint dates to ensure independence between images.

We have seen that the database was created with several constraints in mind. We have limited annual representativeness in order to to limit imbalances within the database. But we have a correct representation of the daily cycle because we have annotated the images by sequence in order to produce images with all the stages in the life of the contrails. Although this database has some limitations, it provides an initial homogeneous database for deep learning using neural networks. The orientation preference of the Sun and the *parasite*s should not impact the learning process if the latter uses a data augmentation implementing rotations in the transformations which is generally the case.

## Ethics Statement

The authors have read and follow the ethical requirement for publication in Data in Brief and confirm that the current work does not involve human subjects, animal experiments, or any data collected from social media platforms. The annotations were made under ethical conditions in-house by the main author with the help from co-authors and the Climaviation team.

## Credit Author Statement

**Nicolas Gourgue:** Methodology, Data curation, Conceptualization, Software, Formal analysis, Writing; **Olivier Boucher:** Supervision, Conceptualization, Writing; **Laurent Barthès:** Supervision, Conceptualization, Writing.

## Data Availability

easydata.earthA dataset of annotated ground-based images for the development of contrail detection algorithms (Original data).

## Appendix Supplementary materials

Image, application 1
