# Supplementary material for: A dataset of annotated ground-based images for the development of contrail detection algorithms
Source: Data Brief. 2025 Feb 4;59:111364. doi: 10.1016/j.dib.2025.111364 (PMC11870224; doi:10.1016/j.dib.2025.111364)
Supplement: Supplementary file 1 [file mmc1.docx]

# Supplementary material


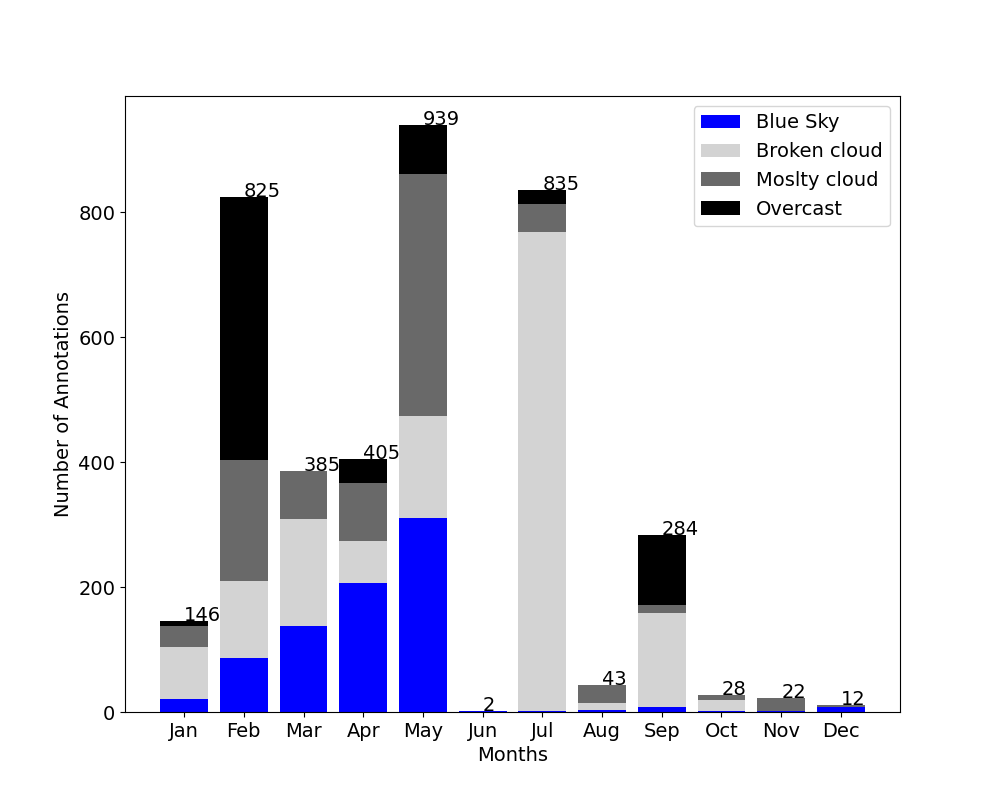


Fig. S1: Histogram of the number of annotations (and its repartition between the four cloud cover tags) as a function of the month in the year.


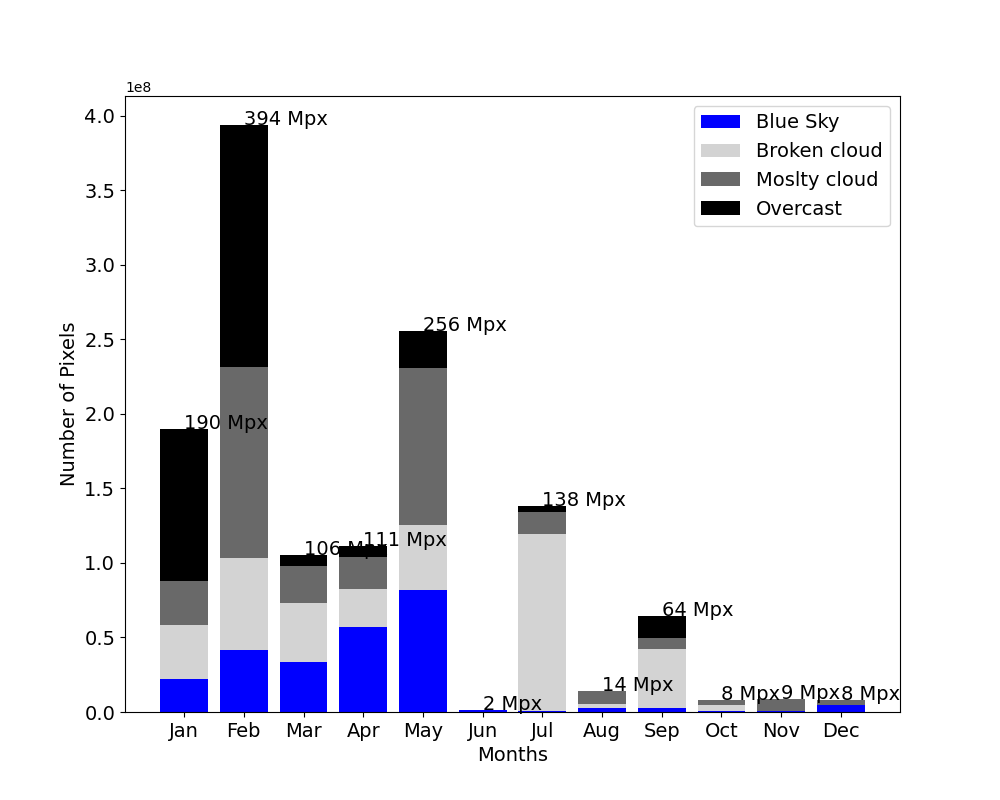


Fig. S2: Histogram of the number of pixels (and its repartition between the four cloud cover tags) as a function of the month in the year.


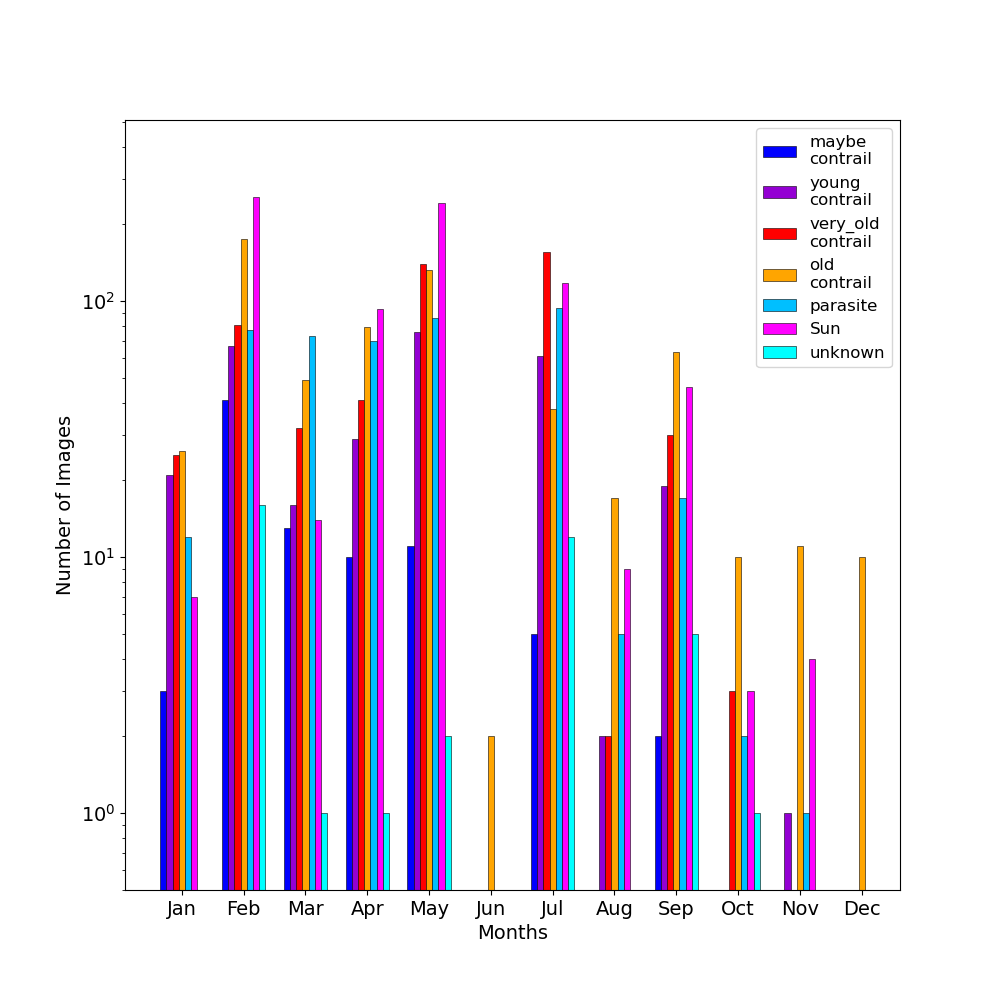


Fig. S3: Histogram of the number of annotations by images class and as a function of the month.


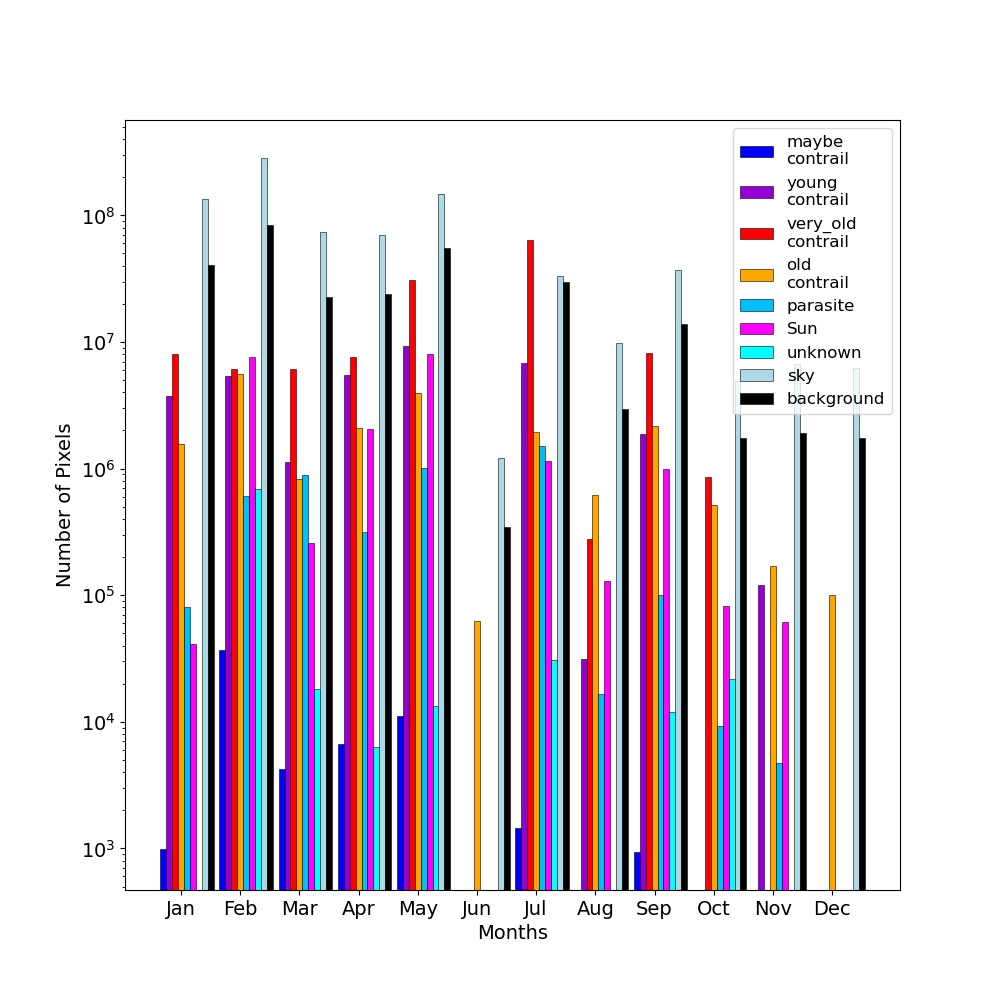


Fig. S4: Histogram of the number of pixels by images class and as a function of the month.


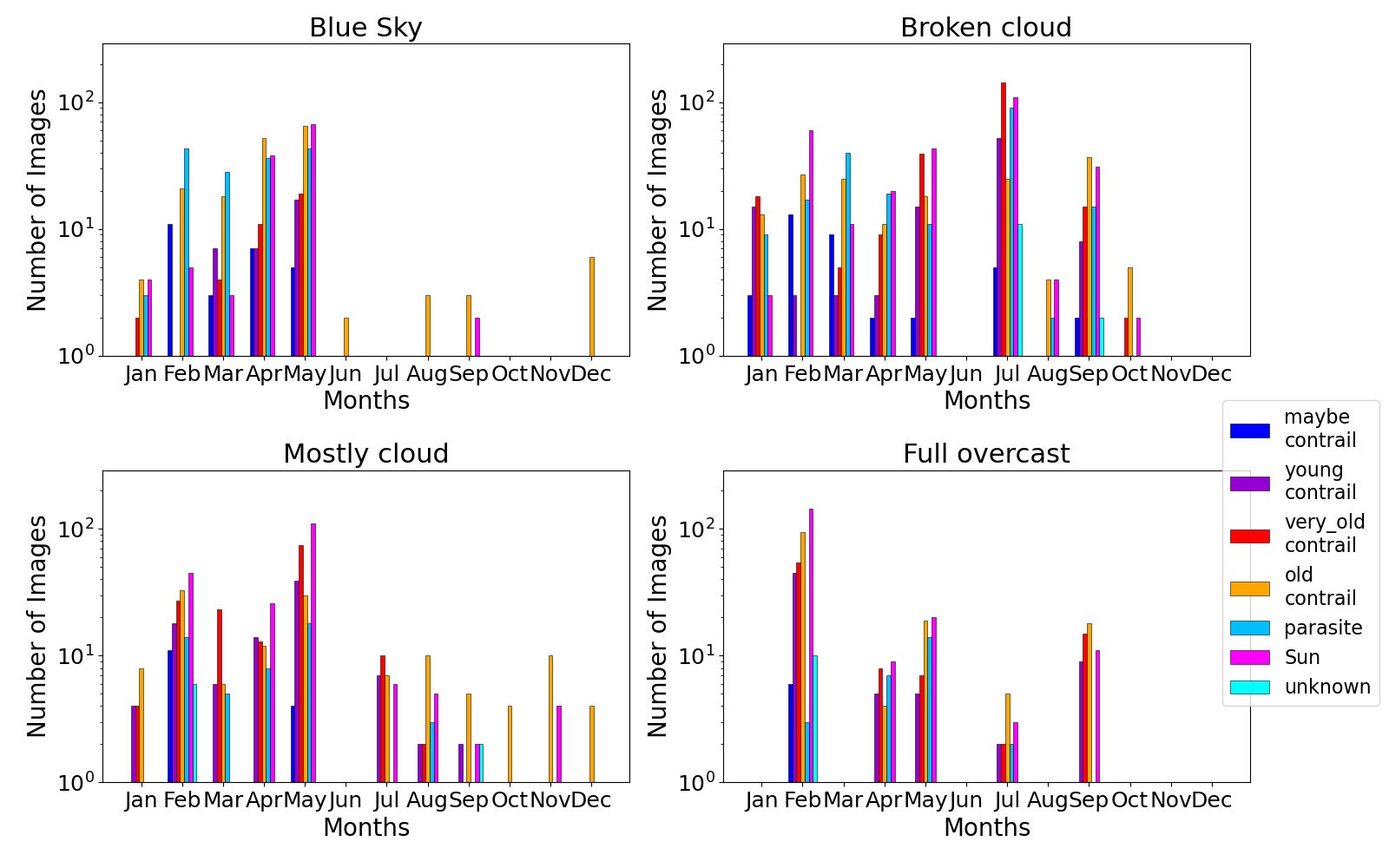


Fig. S5. Repartition of images by class of objects and cloud cover (top left: “blue Sky”; top right: “broken cloud”; bottom left: “mostly cloudy”; bottom right: “full overcast”).


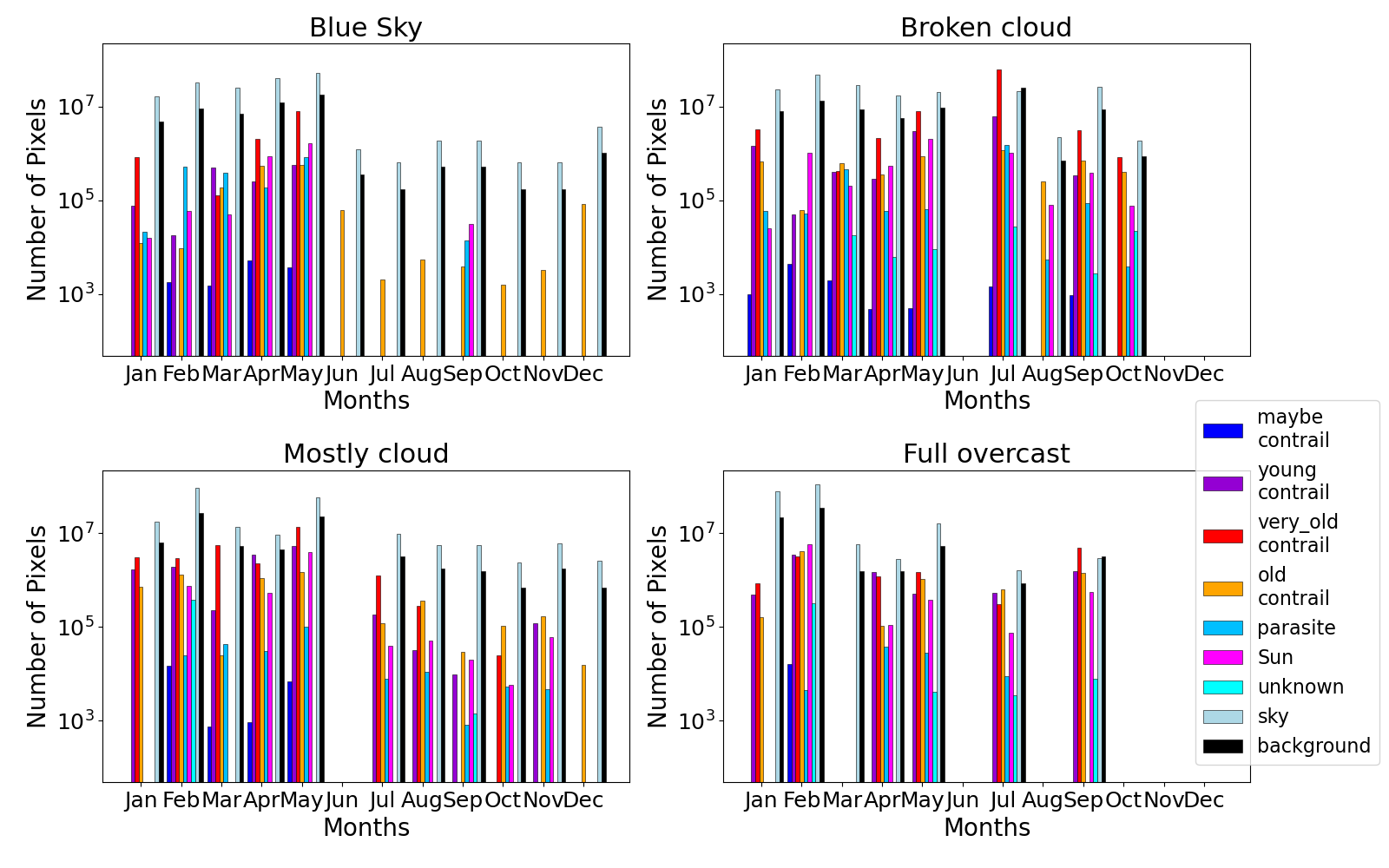


Fig. S.6 Repartition of pixel by class of objects and cloud cover (top left: “blue Sky”; top right: “broken cloud”; bottom left: “mostly cloudy”; bottom right: “full overcast”)


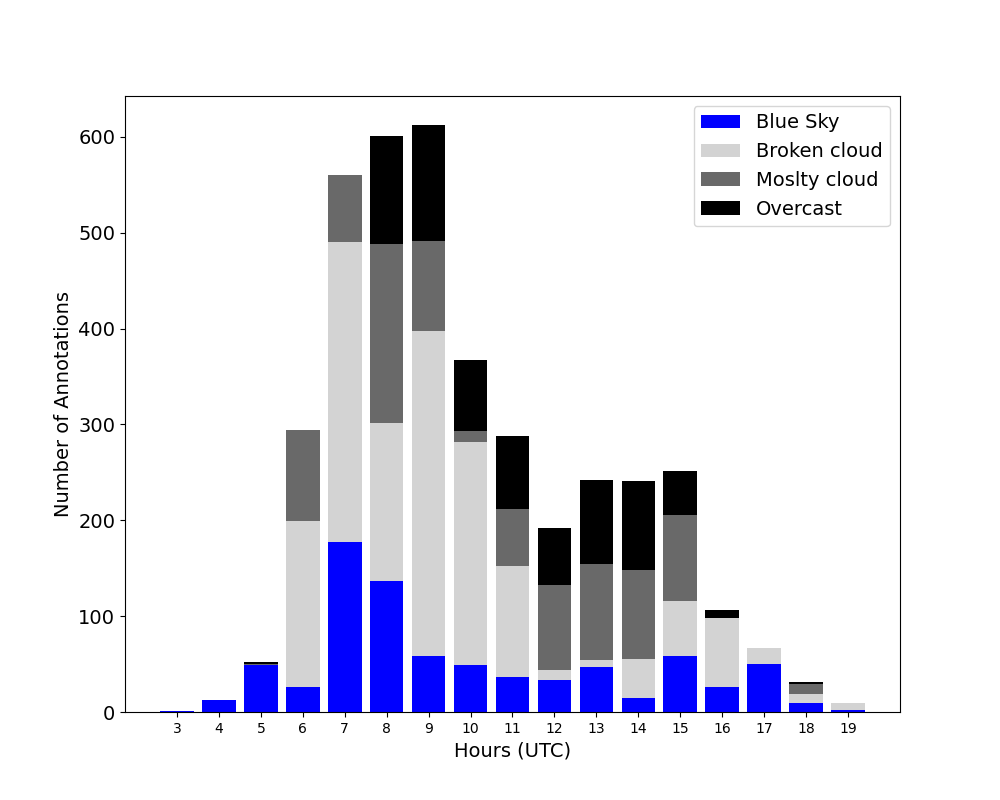


Fig. S7: Histogram of the number of annotations (and its repartition between the four cloud cover tags) as a function of the hours in the day.


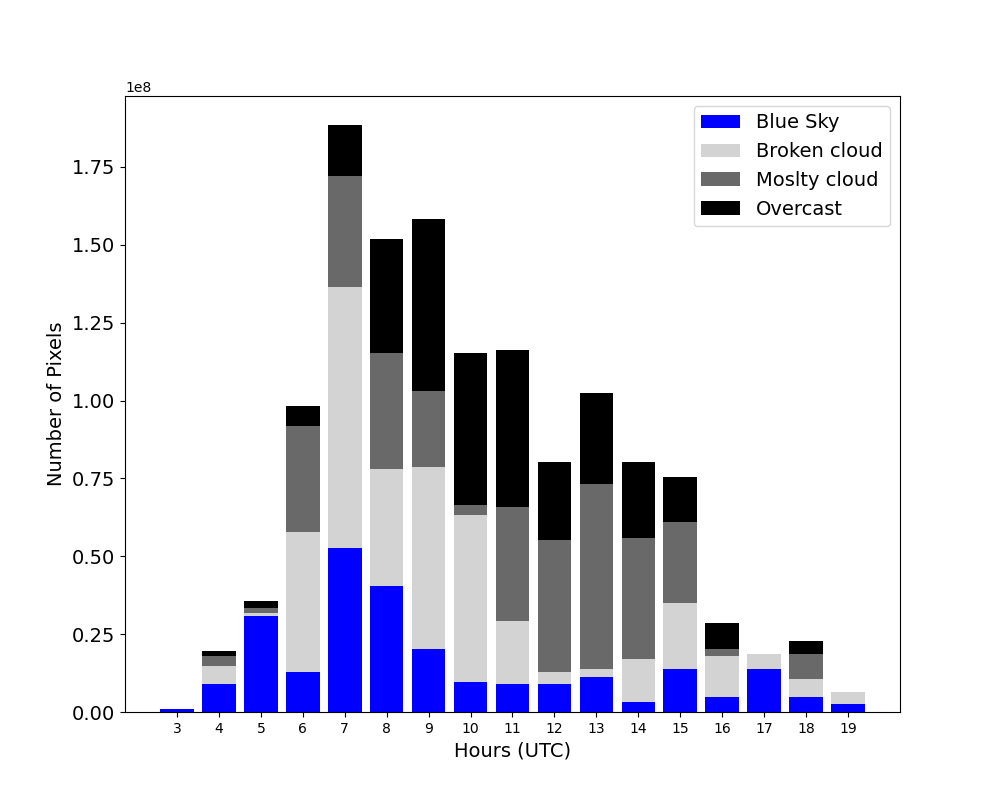


Fig. S8: Histogram of the number of pixels (and its repartition between the four cloud cover tags) as a function of the hours in the day.


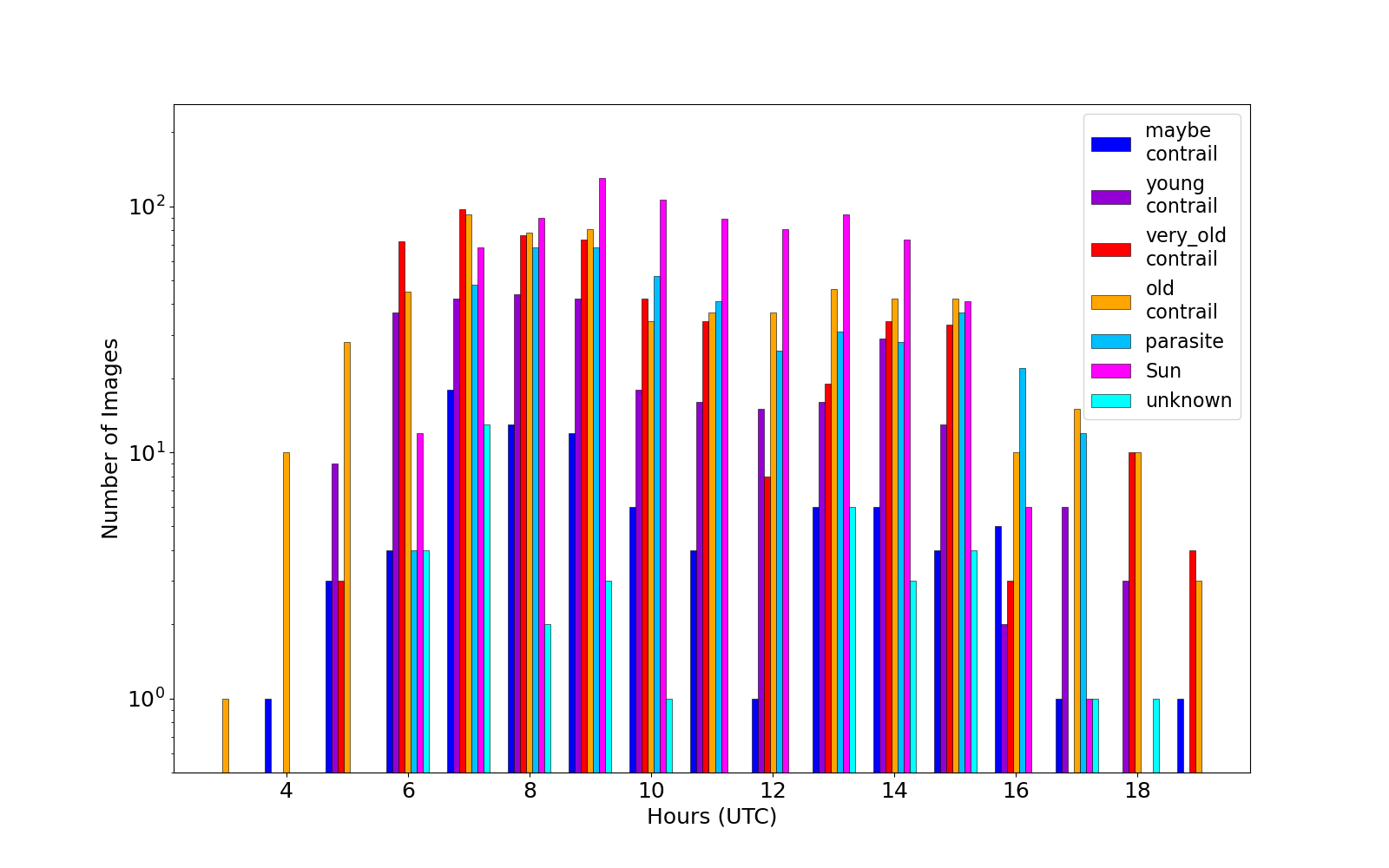


Fig. S9: Histogram of the number of images and its repartition by annotation class as a function of the hour in the day.


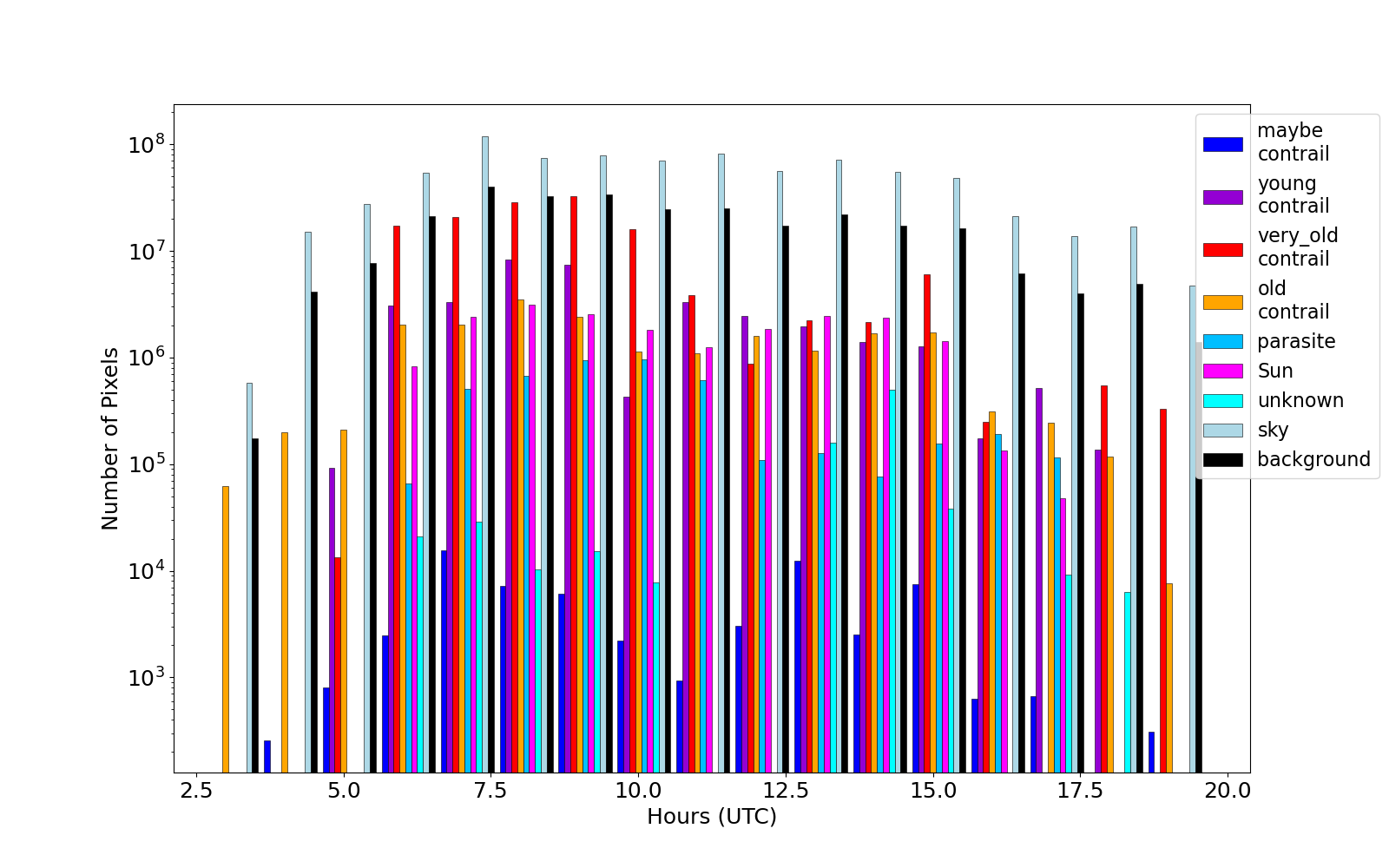


Fig. S10. Histogram of the number of pixels and its repartition by annotation class as a function of the hour in the day.


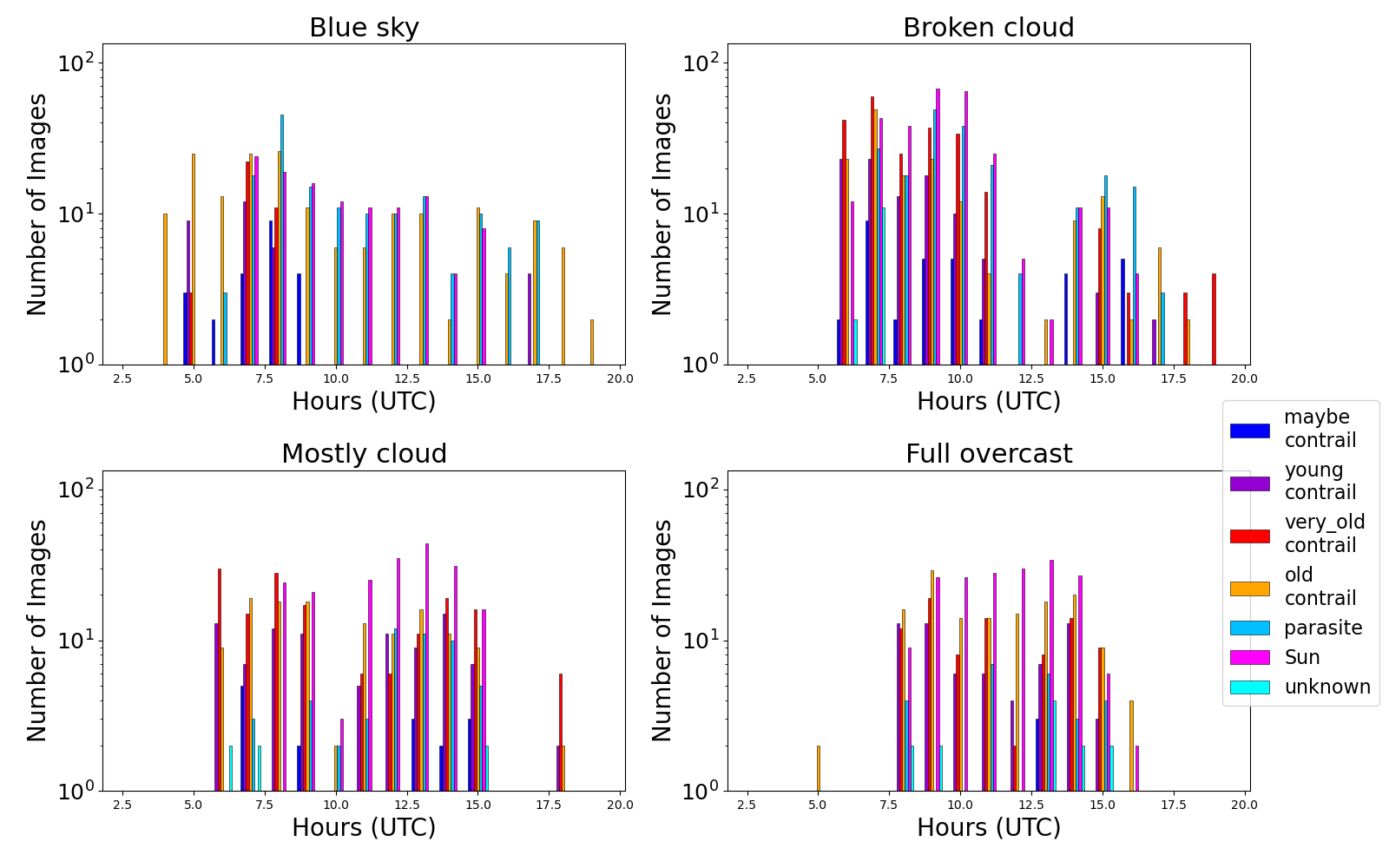


Fig. S11: Repartition of images by class of objects and cloud cover (top left: “blue Sky”; top right: “broken cloud”; bottom left: “mostly cloudy”; bottom right: “full overcast”) by hour with much cloud; top right: class object by hour with full cloudy).


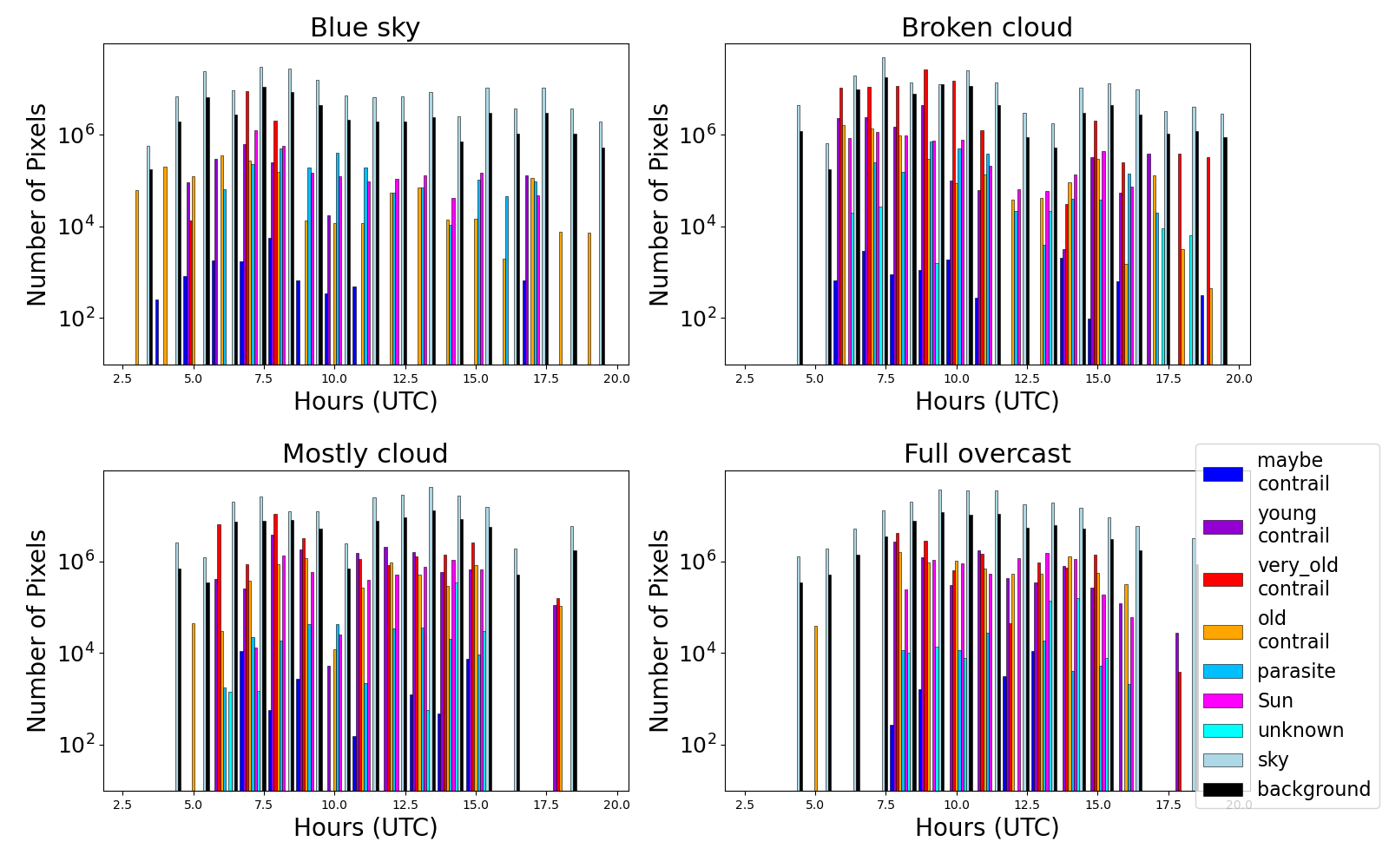


Fig. S12: Repartition of pixel by class of objects and cloud cover (top left: “blue Sky”; top right: “broken cloud”; bottom left: “mostly cloudy”; bottom right: “full overcast”)
